# SCAG-Net: Automated Brain Tumor Prediction from MRI Using Cuttlefish-Optimized Attention-Based Graph Networks

**DOI:** 10.3390/diagnostics16040565

**Published:** 2026-02-13

**Authors:** Vijay Govindarajan, Ashit Kumar Dutta, Amr Yousef, Mohd Anjum, Ali Elrashidi, Sana Shahab

**Affiliations:** 1 Distribution and Supply Technology, Expedia Group, Seattle, WA 98119, USA; vigovindaraja@expediagroup.com; 2 Department of Computer Science and Information Systems, College of Applied Sciences, AlMaarefa University, Riyadh 13713, Saudi Arabia; adotta@um.edu.sa; 3 Research Center, Deanship of Scientific Research and Post-Graduate Studies, AlMaarefa University, Riyadh 13713, Saudi Arabia; 4 Electrical Engineering Department, University of Business and Technology, Jeddah 21432, Saudi Arabia; 5 Engineering Mathematics Department, Alexandria University, Alexandria 21544, Egypt; 6 Department of Computer Engineering, Aligarh Muslim University, Aligarh 202002, India; mohdanjum@zhcet.ac.in; 7 Department of Business Administration, College of Business Administration, Princess Nourah Bint Abdulrahman University, P.O. Box 84428, Riyadh 11671, Saudi Arabia; shahab@pnu.edu.sa

**Keywords:** brain tumor, MRI, attention graph neural networks, Swin-UNet, cuttlefish, segmentation, redundant features

## Abstract

**Background/Objectives:** The earlier, more accurate, and more consistent prediction of the brain tumor recognition process requires automated systems to minimize diagnostic delays and human error. The automated system provides a platform for handling large medical images, speeding up clinical decision-making. However, the existing system is facing difficulties due to the high variability in tumor location, size, and shape, which leads to segmentation complexity. In addition, glioma-related tumors infiltrate the brain tissues, making it challenging to identify the exact tumor region. **Method:** The above-identified research difficulties are overcome by applying the Swin-UNet with cuttlefish-optimized attention-based Graph Neural Networks (SCAG-Net), thereby improving overall brain tumor recognition accuracy. This integrated approach is utilized to address infiltrative gliomas, tumor variability, and feature redundancy issues by improving diagnostic efficiency. Initially, the collected MRI images are processed using the Swin-UNet approach to identify the region, minimizing prediction error robustly. The region’s features are explored utilizing the cuttlefish algorithm, which minimizes redundant features and speeds up classification by improving accuracy. The selected features are further processed using the attention graph network, which handles structural and heterogeneous information across multiple layers, improving classification accuracy compared to existing methods. **Results:** The efficiency of the system, implemented with the help of public datasets such as BRATS 2018, BRATS 2019, BRATS 2020, and Figshare is ensured by the proposed SCAG-Net approach, which achieves maximum recognition accuracy. The proposed system achieved a Dice coefficient of 0.989, an Intersection over Union of 0.969, and a classification accuracy of 0.992. This performance surpassed the most recent benchmark models by margins of 1.0% to 1.8% and with statistically significant differences (p < 0.05). These findings present a statistically validated, computationally efficient, clinically deployable framework. **Conclusions:** The effective analysis of MRI complex structures is used in medical applications and clinical analysis. The proposed SCAG-Net framework significantly improves brain tumor recognition by addressing tumor heterogeneity and infiltrative gliomas using MRI images. The proposed approach provides a robust, efficient, and clinically deployable solution for brain tumor recognition from MRI images, supporting accurate and rapid diagnosis while maintaining expert-level performance.

## 1. Introduction

Recognizing brain tumors [1] is an essential aspect of medical diagnostics, as early detection and proper classification are vital for planning treatment and improving patient survival and quality of life. Whether a brain tumor [2] is malignant or benign, it hinders the central nervous system’s functioning, leading to adverse effects like severe and chronic headaches, seizures, and a combination of memory, speech, and motor coordination impairments [3]. These complications intensely and progressively diminish the patient’s cognitive and physical functions [4], leading to decreased autonomy and quality of life. Additionally, the importance of early detection lies in enabling timely medical intervention; it protects a patient’s ability to undergo effective treatment strategies, including surgery, chemotherapy, and radiotherapy [5], and prevents the tumor from reaching an advanced, inoperable stage.

Early tumor detection [6] greatly enhances treatment efficacy and reduces the risk of patient death. Among multiple diagnostic procedures, Magnetic Resonance Imaging (MRI) [7] is noted for its diagnostic accuracy and as being the least invasive. Unlike CT or PET scans [8], MRI provides greater contrast between soft tissues, helping delineate brain tissue and tumor borders, all without the hazard of ionizing radiation. Predicting brain tumors with MRI involves a series of steps, including preprocessing [9] (noise reduction, skull stripping, and normalization), segmentation (locating tumor-affected areas), feature extraction [10] (texture, intensity, and spatial features), and classification [11,12] (tumor type and degree). Despite this high level of MRI use in diagnosing and informing the treatment of brain tumors, many problems remain. These include glioma cells [13] (tumor forms), which infiltrate brain tissues [14,15] and obfuscate borders; the high variability in the shape, size, and location of the tumor, and the tendency of normal and malignant tissue to overlap in intensity, makes pattern recognition difficult [16]. Additionally, gliomas have dense and compact cellular architectures, so there is a need for accurate localization of the tumor using physical and bioelectrical approaches to destroy tumor cells precisely [17,18].

Current methods, including conventional machine learning approaches and deep learning [19], tend to focus on a narrow research area, leading to a lack of diverse data (generalization), overfitting, and redundant features. The process of manually annotating data for training purposes is time-consuming [20] and may introduce subjectivity. Recent studies seek to mitigate these issues by combining transformer models, attention layers [21], and evolutionary optimization to improve brain tumor recognition accuracy [22,23]. Transformer models have also shown potential in diagnosing gliomas using computational pathology [24]. Many previous studies have demonstrated use of various evolutionary optimization algorithms such as ant colony and swarm intelligence to improve MRI image segmentation for various clinical scenarios [25]. Therefore, integrated models are designed to simultaneously analyze and disentangle the local and global contexts of MRI scans, reduce redundant features, and improve categorization precision. These models provide a reliable and streamlined approach to automating brain tumor identification in diagnostic settings. Therefore, this work introduces the Swin-UNet with cuttlefish-optimized attention-based Graph Neural Networks (SCAG-Net) to improve overall brain tumor recognition accuracy. The effective incorporation of optimization techniques helps address challenges such as tumor heterogeneity and infiltrative gliomas, thereby maximizing overall recognition rates.

The persistent challenges in correctly detecting brain tumors from MRI scans, where significant anatomical variances, overlapping tissue intensities, and intricate tumor boundaries frequently impair the effectiveness of current techniques, are the driving force behind this effort. Current methods usually only maximize one aspect, like feature extraction, segmentation, or classification, which leads to fragmented solutions that do not fully capture the structural and geographic complexity of tumor regions. Furthermore, a lack of annotated data limits the capacity of traditional deep learning models to generalize across patients and imaging circumstances, and high-dimensional MRI features frequently add redundancy. The need for an integrated framework that can simultaneously model global context, improve discriminative features, and reason about structural linkages inside the brain is highlighted by these limitations. The purpose of this study is to address these interrelated problems by developing a single, strong, and clinically accurate architecture for brain tumor prediction.

## 2. Related Works

The most recent work in brain tumor detection from MRI images has focused on refining diagnostic precision, enhancing deep learning frameworks, and improving feature analysis for clinical utility. Zhu et al. (2024) [26] developed an optimized deep neural network and an amended grasshopper-optimization algorithm to refine feature selection and improve tumor classification accuracy. Though this approach effectively minimized overfitting, its applicability to different datasets was somewhat limited. In a similar vein, Daoud et al. (2025) [27] addressed the tumor detection and treatment prediction gap by formulating it within a deep learning framework using the Spider Wasp Optimization algorithm, which offers convergence and interpretability but is computationally intensive for larger datasets. Meenal and Asokan (2026) [28] devised a new quantum-inspired adaptive feature fusion model, motivated by the principles of quantum computing and deep learning, resulting in efficient, highly accurate tumor classification. While their framework efficiently captured the complex features of an MRI, the model’s complexity slowed processing. While Yin and Teng (2026) [29] present the deep fusion framework, which integrates ResNet, EfficientNet, and transformer-based attention mechanisms for robust tumor classification—the model demonstrates impressive generalization across tumor types—it is highly memory-intensive during training. Barati et al. (2025) [30] used lightweight neural networks to analyze the effects of different optimizers and loss functions on the accuracy of brain tumor prediction. Clearly, they demonstrated that the choice of optimizer is critical to achieving high prediction accuracy while keeping the neural network lightweight.

Ullah et al. (2024) [31] developed a multimodal MRI segmentation and classification system using the DeepLabV3+ framework and explainable AI, thereby enhancing the system’s interpretability and the reliability of the diagnosis. Despite these advancements, scalability remained a challenge due to the model’s complexity. For early-stage tumor prediction, Saraswat and Dubey (2025) [32] proposed a dilated, attention-based ensemble network that, in conjunction with enhanced artificial rabbit optimization, achieved high accuracy with reduced false positives, albeit at a high computational cost. Mallouk et al. (2025) [33] introduced the Optimal Deep Transfer Learning (ODTL) model, which improved feature transfer and classification but struggled with domain adaptation to unseen data. Using dual deep convolutional networks, Bernard et al. (2025) [34] improved feature extraction from MRI scans, thereby increasing the precision of tumor localization and classification. However, this paradigm was limited by the requirement of extensive annotated datasets for practical model training. Additionally, in recent times, emerging sequence modeling architectures such as Mamba have also been successfully utilized for medical image segmentation with strong capability in capturing long-range dependencies under limited data [35]. Hybrid models that integrate convolutional neural networks for region localization and transformer-based components for global dependencies have demonstrated remarkable outcomes for various MRI related clinical task such as brain tumor segmentation from MRI images [36] and long-term outcome prediction from cardiac MRI [37].

On the other hand, not dissimilar to the contribution mentioned above, Hasan et al. (2025) [38] introduced DEEP Q-NAS, a revision of neural architectures that implements reinforcement learning to automate the process of designing deep networks for tumor identification, achieving considerable adaptability and accuracy, albeit with substantial training time and high computational cost. The works mentioned above provide a trajectory of the development of hybrid, optimized, and interpretable frameworks, with deep learning still, relative to the other branches of AI, suffering from a lack of integrated models that fuse the precision of diagnostics with the rapidity of computation and clinical relevance, a deficit that SCAG-Net aims to address. According to various researchers’ work, the findings are summarized in Table 1.

Recent pertinent studies on MRI-based brain tumor identification, such as Zhu et al. (2024) [26], Daoud et al. (2025) [27], Meenal and Asokan (2026) [28], Yin & Teng (2026) [29], and Hasan et al. (2025) [38], have been compared with the SCAG-Net design. The shortcomings of earlier models include their low clinical scalability, limited generalization, high computing cost, and isolated module optimization. The comparison research shows that new brain tumor recognition models use a mix of deep learning and optimization techniques to achieve very accurate results, but they often make computations less efficient and harder to understand. Transfer learning, attention fusion, and evolutionary optimization are all excellent methods for generalization and scaling, but most of them do not work well in clinical settings or in real time. The results show that we need a balanced framework, such as the proposed SCAG-Net, to ensure that medical tests are accurate, efficient, and reliable for real-world use.

Despite the high classification accuracy reported by recent MRI-based brain tumor detection studies using transformers, evolutionary optimization, or attention mechanisms, Table 1 shows that these methods primarily function as separate or loosely coupled modules, which results in limitations in clinical scalability, computational efficiency, and uncertainty propagation. The correlation between accurate tumor localization and final diagnostic confidence is weakened in many modern works, particularly in infiltrative gliomas with ambiguous boundaries, because optimization is only applied at the classifier level or attention is used without explicitly preserving structural dependencies between segmentation and recognition. Because of this, performance improvements in one step frequently do not translate to the entire diagnostic pipeline. This disparity emphasizes the necessity of an integrated model that tightly integrates structural reasoning, feature refining, and segmentation into a single framework. Such an integrated approach can mitigate error accumulation, enhance robustness across heterogeneous MRI modalities, and improve clinical reliability by jointly modeling spatial context, removing redundant features before classification, and maintaining inter-regional relationships through graph attention. Therefore, the proposed SCAG-Net framework has been designed.

## 3. SCAG-Net Framework for Brain Tumor Detection

### 3.1. Problem Definition

Automatic brain tumor detection from MRI is affected by a high-dimensional, multivariate prediction problem due to intra-class variability, feature redundancy, and spatial irregularities. Consider $D=\{\delta_{1},\delta_{2},\ldots ,\delta_{N}\}$ is the MRI brain image in which every $\delta_{i}$ related to channel intensities is distributed across different coordinates. These instances $\delta_{i}$ are linked with the labels $\lambda_{i}\in \{\phi_{1},\phi_{2},\ldots ,\phi_{K}\}$ that are related to the healthy region or tumor (K). The $\delta_{i}$ related latent variables are obtained by applying the mapping Ψ:D→Λ in which the prediction process is defined as ${\hat{\lambda }}_{i}=\Psi (\delta_{i})$ that faces the risk called generalization loss $R(\Psi )=E_{\left(\delta ,\lambda \right)}[L(\Psi (\delta ),\lambda )]$ because it creates misclassification issues. The Ψ process was affected by several factors, including heterogeneity in brain structure, glioma infiltration, dimensionality, and data imbalance. For every patient, the brain structure is changed in its intensity distribution and has the non-uniform boundaries that create difficulties while separating the non-linearity between the abnormal and normal tissues in the feature space $\left(f_{s}\right)$. Then, the glioma infiltration creates the longitudinal overlap between the healthy voxel $\left(h_{v}\right)$ and pathological voxel $\left(P_{v}\right)$, which causes uncertainty issues when predicting exact decision boundaries. The third significant problem occurs due to the MRI’s dimensionality, in which every observation $\delta_{i}$ comes under the $R^{u\times v\times w\times m}$ high-order tensor domain that leads to irrelevant or redundant features, reducing the overall efficiency of brain tumor prediction. Finally, data imbalance leads to biased classification decisions and reduces overall brain tumor recognition accuracy, resulting in a high misclassification error rate. Therefore, the brain tumor system requires trained and learned systems ($\Psi^{*}$) for handling the high-dimensional, noisy, and spatially consistent MRI data to minimize the redundant attributes and uncertainty issues effectively.

### 3.2. Research Contribution

The objective of this work is to create the Swin-UNet with the cuttlefish-optimized attention graph network (SCAG-Net) to achieve generalized and robust brain tumor prediction systems. The developed SCAG-Net framework intends to reduce the classification error rate and uncertainty issues while performing $\Psi^{*}$, which uses discriminative learning to predict brain tumors. This objective is achieved by applying the hierarchical structural encoding that observes the local and global contextual dependencies from D using the $\Phi_{\mathrm{seg}}$. The mapping-related encoding process maximizes the $f_{s}$ by updating the $\underset{\Phi_{\mathrm{seg}}}{max\;}\;\Omega_{1}=\frac{\sum_{t=1}^{T}∥\nabla f_{t}{∥}_{2}^{2}}{T}$. The mapping process confirms that high-frequency anatomical changes, such as structural boundaries and tumor edges, are handled during encoding, thereby minimizing spatial ambiguity. Then, the derived $F_{h}$ are processed with the help of the cuttlefish optimization techniques to refine the feature ($\Xi_{\mathrm{opt}}$) that reduces the redundant attributes. In addition, the $\Xi_{\mathrm{opt}}$ process increases the discriminative separability by maximizing the inter-class dispersion and reducing the intra-class variances, which are defined as $\underset{\omega \in \Omega }{min\;}\;J(\omega )=\alpha \;{\mathrm{Var}}_{\mathrm{intra}}(F_{\omega })-\beta \;{\mathrm{Var}}_{\mathrm{inter}}(F_{\omega })$. The effective utilization of the $\rho_{r}$ and $\rho_{v}$ chromatophore behavior helps to identify the optimal subset feature $F_{o}=\Xi_{\mathrm{opt}}(F_{h})$ that maximizes the discriminative capacity and reduces the redundancy successfully. In addition, the selected $F_{o}$ is processed by an attention mechanism ($\Gamma_{\mathrm{attn}}$) to identify the structural relationship by constructing the graph (G=(V,E)), which reduces the reconstruction error ($\underset{\Gamma_{\mathrm{attn}}}{min\;}\;\Omega_{2}=∥L-\hat{L}{∥}_{F}^{2},$) to guarantee the learned embedding $\left(e_{i}\right)$ to manage the brain’s operational geometry. At last, global integration and optimization are applied to obtain a high classification confidence value, which is defined as $\underset{\Psi^{*}}{min\;}\;R_{\mathrm{total}}=\lambda_{1}L_{\mathrm{seg}}+\lambda_{2}L_{\mathrm{opt}}+\lambda_{3}L_{\mathrm{graph}}$ which also reduces the loss value $\Psi^{*}(\delta )=\Gamma_{\mathrm{attn}}(\Xi_{\mathrm{opt}}(\Phi_{\mathrm{seg}}(\delta )))$ and ensures the robustness and topological consistency across different MRI inputs.

To improve the logical continuity of the proposed methodology, the variables introduced across preprocessing, segmentation, optimization, and classification stages are explicitly linked through a unified mathematical formulation. Let the multimodal MRI input be denoted as $X\in R^{\left(H\times W\times D\right)}$, where H, W, and D represent spatial dimensions and M denotes the number of imaging modalities. The preprocessing operator $P\left(\cdot \right)$ transforms X into a normalized volume $X^{\prime }=P\left(X\right)$, which is then mapped by the Swin-UNet encoder–decoder $S\left(\cdot ;\theta_{s}\right)$ to produce both a segmentation mask $\hat{Y}=S\left(X^{\prime };\theta_{s}\right)$ and a corresponding feature set $F=\left\{f_{1},f_{1},\ldots ,f_{N}\right\}$. To reduce feature redundancy, cuttlefish optimization learns a binary selection vector $b\in {\left\{0,1\right\}}^{N}$, yielding refined features $F^{⋆}=F⊙b$. These optimized features are subsequently embedded into a graph $G=\left(V,E\right)$, where each node is parameterized by $F^{⋆}$ and edges encode spatial and structural similarity. Finally, an attention-based graph network $G\left(\cdot ;\theta_{g}\right)$ operates on this graph to estimate the tumor class label $\hat{c}=G\left(G;\theta_{g}\right)$. This explicit functional chaining clarifies variable dependencies and ensures coherent information flow across all stages of the SCAG-Net framework.

### 3.3. SCAG-Net Framework

**Figure 1:**
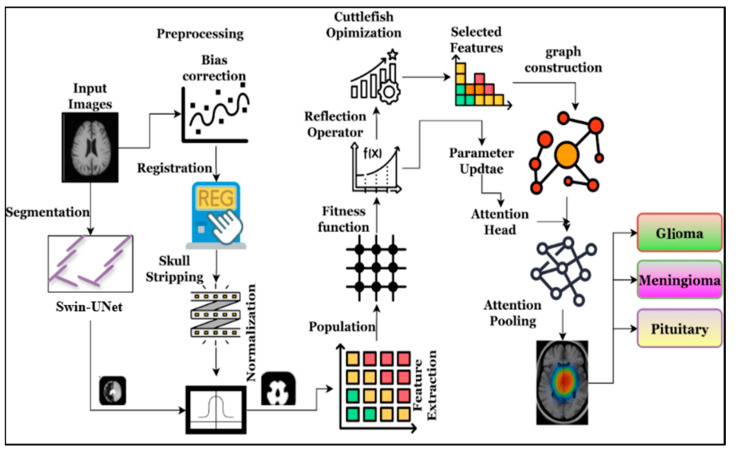
Overall architecture of SCAG-Net framework for multimodal brain MRI analysis.

This research presents the SCAG-Net framework, which aims to provide an intelligent and dependable system for accurate, timely brain tumor prediction from MRI. The work seeks to address the difficulties that deep learning models face with feature redundancy, structural inconsistency across MRI modalities, tumor variability, and a lack of confidence in existing models. With multi-scale spatial encoding and iterative feature refinement via a graph attention mechanism, SCAG-Net incorporates tractable local textures and organizational structures for a comprehensive within-region analysis of the brain. Therefore, the SCAG-Net deep learning framework has received unprecedented attention for providing a clinically useful system that augments diagnostic accuracy while maintaining minimal computational cost and consistently delivering the required tasks across heterogeneous medical datasets. The research objective is achieved via a step-by-step process is described in Figure 1.

Figure 1 shows the overall workflow of the SCAG-Net framework for automatically identifying brain tumors from MRI images. The framework starts with a preprocessing step that performs functions such as skull stripping and normalization. Then the Swin-UNet is applied to obtain multi-dimensional features, which are processed by cuttlefish optimization to refine and eliminate redundant features from the feature set. Finally, the attention-based graph network is applied to identify brain tumors by understanding brain structure, thereby helping predict the output label while addressing uncertainty and improving clinical reliability.

#### 3.3.1. Multimodal MRI Preparation and Preprocessing

**Figure 2:**
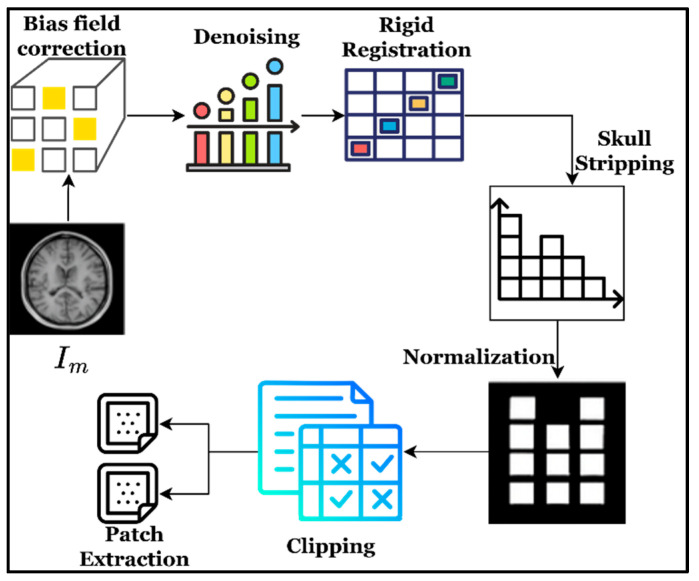
Process of MRI image preparation and preprocessing.

The first step of this work is to explore the heterogeneous D for obtaining the model-ready, consistent, and clean image to improve the overall brain tumor recognition accuracy. The preparation and processing phases effectively eliminate noise, variability, alignment artifacts, non-brain voxels, and problems with intensity distribution. As said, $D=\{\delta_{1},\ldots ,\delta_{N}\}$ has several instances, and every patient $\left(\delta_{i}\right)$ has 4-channel tensors that are represented as $\delta_{i}=[M_{i}^{\left(1\right)},M_{i}^{\left(2\right)},M_{i}^{\left(3\right)},M_{i}^{\left(4\right)}]\in R^{H\times W\times D\times 4}$ which are relevant to the $T_{1},\;T_{2},\;T_{1ce}\;and\;FLAIR$; each $\delta_{i}$ has its own width $\left(W\right)$, height $\left(H\right)$, and depth $\left(D\right)$. The gathered $\delta_{i}$ is processed through a sequence of steps to improve image quality and the efficiency of brain tumor recognition, as shown in Figure 2.

As shown in Figure 2, the collected MRI images exhibit a low-frequency intensity bias, requiring immediate action to improve overall brain structure analysis. Therefore, the equivalent bias correction is applied to each model ${\tilde{M}}_{i}^{\left(k\right)}=N_{4}\left(M_{i}^{\left(k\right)}\right),$ in which image intensity is approximately factors (M=B⋅S+η) and computes the bias field (B) that enhances the overall skull stripping and normalization. Then, every model is further explored using block matching, in which the non-local mean value (${\bar{M}}_{i}^{\left(k\right)}=\mathrm{NLM}({\tilde{M}}_{i}^{\left(k\right)}))$ is estimated by computing the weighted average value of the intensity or color pixel in the image. The weight $\left(w_{i}\right)$ is identified by the similarity between the patches $\left(p_{i}\right)$. The non-local mean-based computations minimize the local $p_{i}$ variations ($\mathrm{Var}\;\left(\cdot \right)$) while managing the anatomical information. Then, computed ${\bar{M}}_{i}^{\left(k\right)}$ are aligned to identify the reference generally $T_{1}\;and\;T_{1ce}$; rigid transformation $\left(T_{i}\right)$ is then estimated with the help of the mutual information $M_{i}^{(k)\prime }(x)=M_{i}^{\left(k\right)}(T_{i}(x)),\forall k,$ which gives the registered tensor value $\left(\delta_{i}^{\prime }\right)$. As said before, the gathered images in D have different voxel sizes that require resampling to minimize the size $s^{*}=(s_{x}^{*},s_{y}^{*},s_{z}^{*})$ which is accomplished with the help of linear operations, and the resampling is represented as $M_{i}^{(k)*}=\mathrm{Resample}\left(M_{i}^{{\left(k\right)}^{\prime }},s^{*}\right)$ which produces the consistent dimension images $M_{i}^{(k)*}\in R^{H^{*}\times W^{*}\times D^{*}}$. Afterwards, the resampled brain images are frequently explored to obtain the brain mask $B_{i}\in \{0,1{\}}^{H^{*}\times W^{*}\times D^{*}}$ by applying the Swin-Unet that is discussed in Section 3.3.2, which is combined with the modality by performing the element-wise operation (⊙) that eliminates the background voxel, scalp, and skull successfully. Then, z-score normalization is applied to convert the images into the standardized format that is defined in Equation (1).

(1) $$\left.\begin{array}{c} \begin{array}{c} \mu_{i}^{\left(k\right)}=\frac{1}{|B_{i}|}\sum_{x:\;B_{i}\left(x\right)=1}S_{i}^{\left(k\right)}\left(x\right),\\ \sigma_{i}^{\left(k\right)}=\sqrt{\frac{1}{|B_{i}|}\sum_{x:\;B_{i}\left(x\right)=1}(S_{i}^{\left(k\right)}(x)-\mu_{i}^{\left(k\right)}{)}^{2}} \end{array}\\ {\hat{M}}_{i}^{\left(k\right)}(x)=\frac{S_{i}^{\left(k\right)}(x)-\mu_{i}^{\left(k\right)}}{\sigma_{i}^{\left(k\right)}}for\;B_{i}(x)=1. \end{array}\right\}$$

The normalized modality $\left({\hat{M}}_{i}^{\left(k\right)}(x)\right)$ is computed from the statistical computation of the binary mask $\mu_{i}^{\left(k\right)}\;and\;\sigma_{i}^{\left(k\right)}$. Again, the outliers are removed by estimating the clip intensities, such as $p_{\mathrm{low}},p_{\mathrm{high}}$ in which the brain voxels are rescaled to [0,1], which is achieved by using Equation (2).

(2) $$\left.\begin{array}{c} {\hat{M}}_{i}^{\left(k\right)}\leftarrow clip\left({\hat{M}}_{i}^{\left(k\right)},q_{1\%},q_{99\%}\right),\\ {\hat{M}}_{i}^{\left(k\right)}\leftarrow \frac{{\hat{M}}_{i}^{\left(k\right)}-min\;}{max\;-min\;}. \end{array}\right\}$$

The rescaled images are explored using spatial cropping in which tight bounding boxes are utilized to examine the brain mask ($B_{i}$) with fixed size $H_{0}\times W_{0}\times D_{0}$ to minimize the empty background that is defined as $\mathrm{bbox}=\mathrm{BoundingBox}(B_{i}),{\tilde{M}}_{i}^{\left(k\right)}={\hat{M}}_{i}^{\left(k\right)}[\mathrm{bbox}]$. Finally, data augmentation, like rotation (R), random flips (F), jitter (J), and elastic deformation (E) is performed, which is denoted as Augment (M)=J(B(E(R(M)))). The above-discussed process provides complete, consistent, noise-free MRI images that help increase the accuracy of brain tumor recognition. The pseudocode for this phase is described in Algorithm 1.
**Algorithm 1:** Pseudocode for MRI data preparation and processing
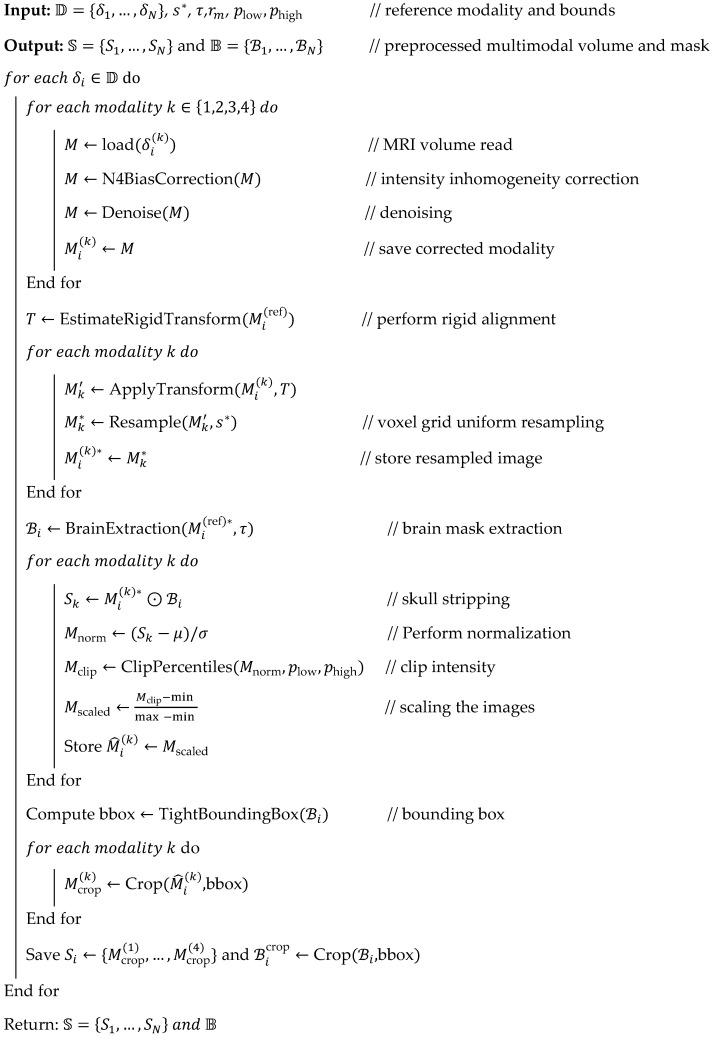


**Figure 3:**
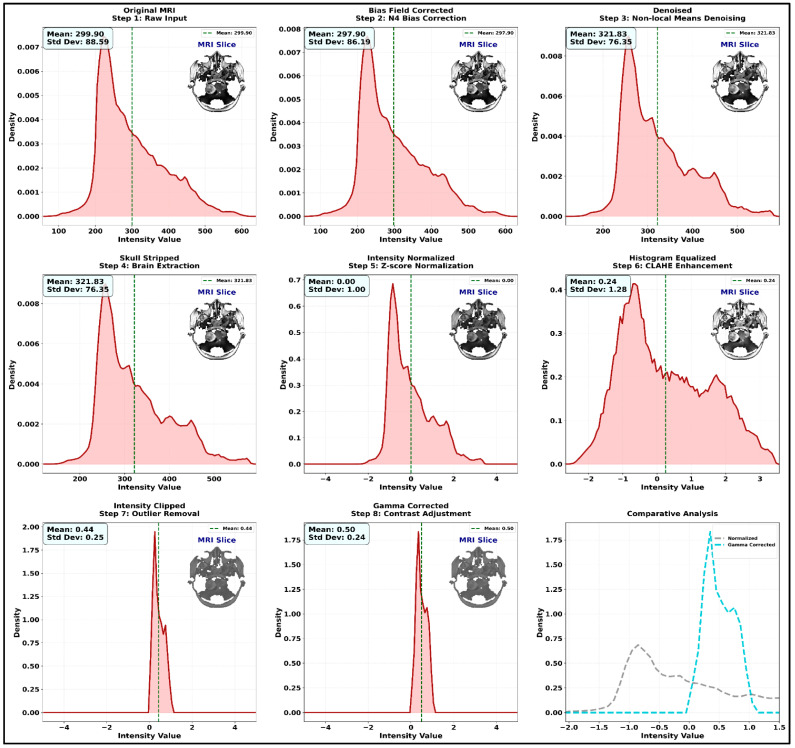
Stage-wise image and histogram analysis of brain MRI images.

According to Algorithm 1, the incoming images are processed to ensure modality alignment, effectively address bias field and inhomogeneity issues, and manage brain tissues while retaining the brain structure. In addition, the resampling and normalization process provides consistent intensity and uniform voxel spacing for patients, enabling the effective attainment of structurally aligned, balanced, and clean MRI images. The above step-based MRI images are widely used for subsequent segmentation and classification. The respective results and histogram representation of each step involved in the preprocessing are shown in Figure 3.

#### 3.3.2. Swin-UNet-Based Binary Mask and Feature Extraction

**Figure 4:**
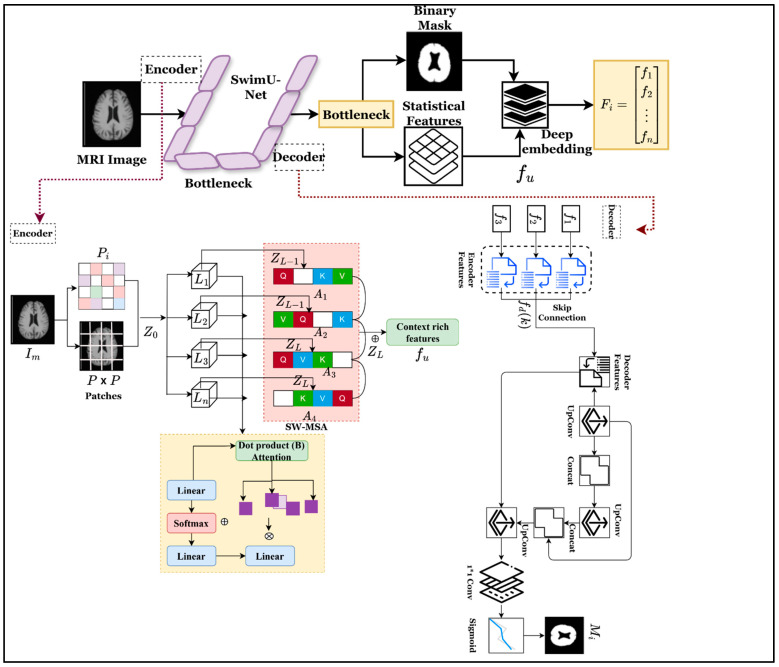
Process of encoder–decoder-based segmentation mask ($M_{i}$) and feature extraction ($f_{u}$) on brain image ($I_{m}$).

The obtained S from the preprocessing image only consists of brain tissue details that help to identify the brain mask region and features effectively. As mentioned, the previous section identified the true brain region and tumor-affected regions using the Swin-UNet segmentation model, which successfully identified these regions by analyzing the images. The extracted $B_{i}$ preserves brain anatomical structures and boundaries with high-pixel precision, and region-level embeddings are widely used to identify the exact brain tumor with maximum prediction accuracy. During analysis, a Swin transformer encoder captures global contextual information via a shifted-window self-attention mechanism, and a U-shaped decoder reconstructs the spatial map to identify the exact binary mask. Consider the preprocessed image is $X_{i}$ with a size of H×W×C that is fed into the Swin network function ($F_{\theta }$) which gives the soft mask, which is defined as ${\hat{Y}}_{i}=F_{\theta }(X_{i})\in [0,1{]}^{H\times W}$. In this mask, ${\hat{Y}}_{i}(x,y)$ represents the pixel wise probability indicating whether a given pixel belongs to a brain tumor. The overall process of binary mask prediction and feature extraction is shown in Figure 4.

As shown in Figure 4, the encoder divides the $X_{i}$ into the non-overlapping patches with P×P size which is represented as $Z_{0}=\mathrm{PatchEmbed}(X_{i})$. During this process, the shifted-window self-attention approach and layer normalization are used to obtain the patches defined in Equation (3).

(3) $$\left.\begin{array}{c} Z_{l}=SW\text{-}MSA\left(\mathrm{LN}\left(Z_{l-1}\right)\right)+Z_{l-1},l=1,\ldots ,L\\ {\mathrm{Attention}}_{h}(Q,K,V)=Softmax(\frac{Q_{h}K_{h}^{⊤}}{\sqrt{d_{h}}}+B_{h})V_{h} \end{array}\right\}$$

In Equation (3), the attention mechanism uses the values ($V_{h}$), key ($K_{h}$), and query ($Q_{h}$) metrics are utilized to obtain the linear projection and position bias ($Z_{l-1}\;and\;B_{h}$). The multi-head attention outputs are combined to obtain rich image features. Then, spatial up-sampling is performed using a decoder to reconstruct the segmentation map that is defined as $F_{d}^{\left(k\right)}=\mathrm{UpConv}(\mathrm{Concat}(F_{e}^{\left(k\right)},F_{d}^{\left(k+1\right)}))$. Here, the encoder feature depth ($F_{e}^{\left(k\right)}$) and skip connections are utilized to prevent the localized information that is lost at the time of down-sampling. The output mask is obtained by applying the 1 × 1 convolution with a sigmoid function and trainable parameters ($W_{o}\;and\;b_{o})$ which is represented as ${\hat{Y}}_{i}=\sigma (W_{o}*F_{d}^{\left(1\right)}+b_{o})$. The identified segmentation map is applied to compare with the threshold value to determine the binary mask, and the condition is defined as $M_{i}(x,y)=\left\{\begin{array}{cc} 1, & \mathrm{if}\;{\hat{Y}}_{i}(x,y)\geq \tau \\ 0, & \mathrm{otherwise} \end{array}\right.\;\mathrm{where}\;\tau \in [0.4,0.6]$. The identified regions ($M_{i}(x,y))$ are applied to the morphological process ($M_{i}^{\prime }=\mathrm{Close}(\mathrm{Erode}(M_{i})))$ to be further refined for improving the brain tumor recognition process, and the resulting binary mask is denoted as $M_{i}^{\prime }$ which consists of several spatial constraints for attribute extraction. The features are extracted from $M_{i}^{\prime }$ in which each patch u has a set of feature vectors that is denoted as $f_{u}\;$, and the derived features are shown in Equation (4).

(4) $$\left.\begin{array}{c} f_{u}=[\;f_{stat}(u)\;||\;f_{tex}(u)\;||\;f_{deep}(u)\;]\\ f_{stat}(u)=[\mu_{u},\sigma_{u},{\mathrm{skew}}_{u},{\mathrm{kurt}}_{u}]\\ \begin{array}{c} f_{tex}(u)=[\mathrm{Energy},\mathrm{Contrast},\mathrm{Entropy},\mathrm{Homogeneity},{\mathrm{LBP}}_{hist}]\\ F_{deep}^{\left(i\right)}=\mathrm{GlobalAvgPool}(Z_{L}) \end{array} \end{array}\right\}$$

According to Equation (4), the features are extracted, which are represented as $F_{i}=[f_{1};f_{2};\ldots \;;f_{n}]\in R^{n\times d}$, and the features consist of semantic and contextual information about brain tissues. During this analysis, the Swin-UNet training is utilized to reduce the segmentation loss $L_{seg}=\lambda_{1}(1-\mathrm{Dice}(\hat{Y},Y))+\lambda_{2}\mathrm{BCE}(\hat{Y},Y)$ and improve the prediction process $\theta^{*}=arg\;\underset{\theta }{min\;}\;E_{(X,Y)∼D}[L_{seg}(X,Y;\theta )]$ for all MRI images. The respective process is shown in Algorithm 2.
**Algorithm 2:** Pseudocode for binary mask and feature extraction**
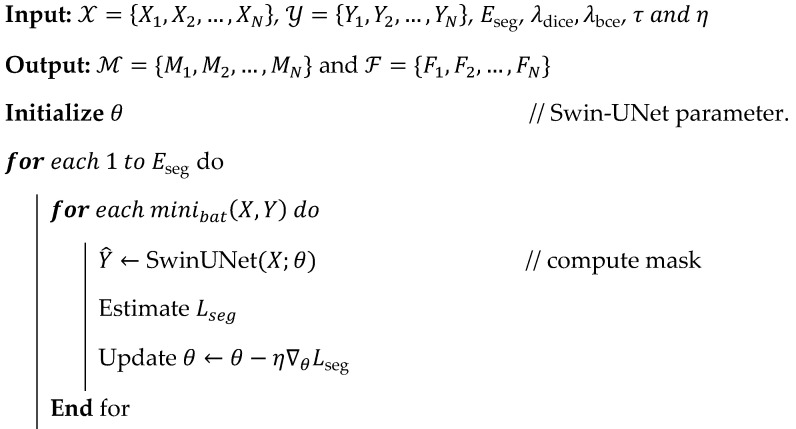

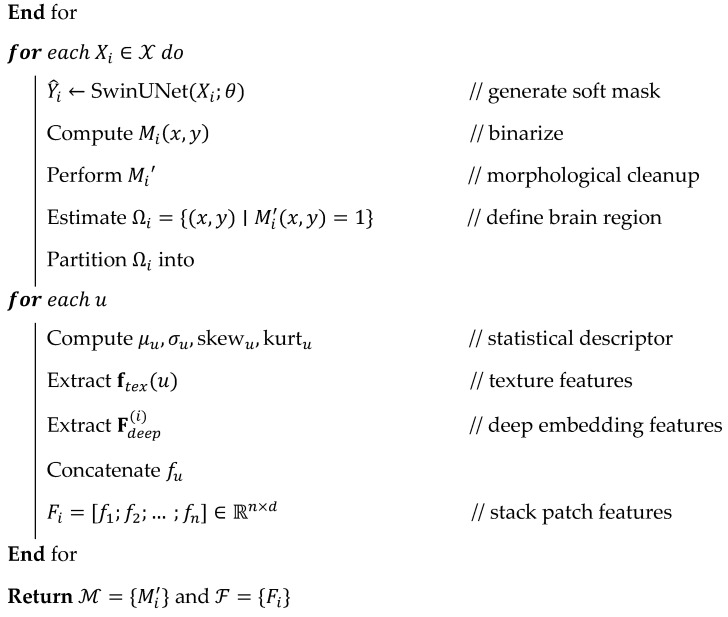
**

**Figure 5:**
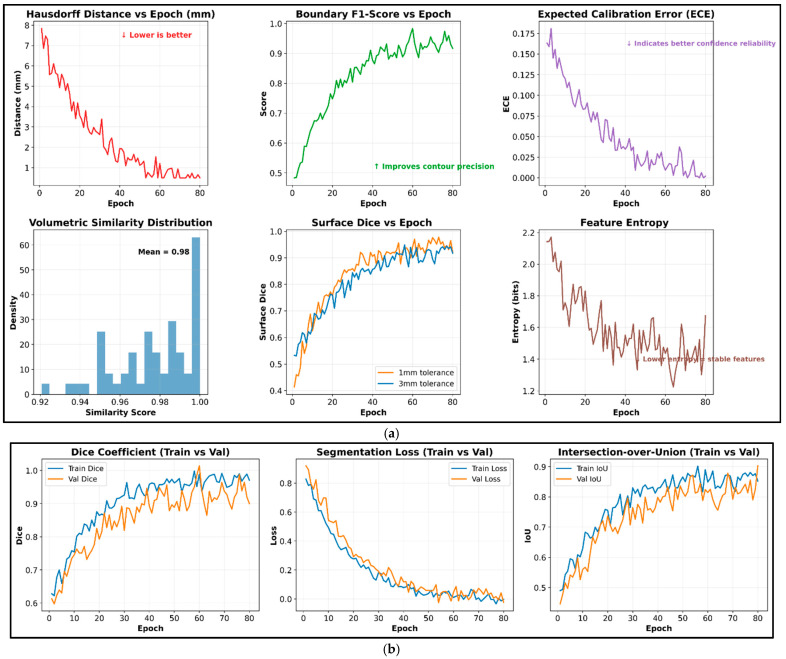
Phase 2 evaluation of brain tumor MRI mask and feature extraction analysis. (**a**) Quantitative analysis of Swin-UNet-based brain mask derivation and feature extraction analysis for various numbers of epochs. (**b**) Evaluation of Swin-Unet for training and validation datasets.

The above-discussed Algorithm 2 effectively captures the brain’s structural information using the Swin-UNet segmentation and feature extraction process. During analysis, the encoder and decoder processes eliminate irrelevant details, such as background tissues and skull information, to improve overall contextual analysis efficiency. In addition, the Swin-UNet uses multiple layers that extract statistical texture and deep embeddings that identify entire statistical descriptors, thereby directly improving overall brain tumor prediction efficiency. The derived embeddings are consistent and effectively capture the intrinsic variations in morphology, structure, and intensity. The extracted features are fed into the following feature selection and classification process to improve the overall brain tumor identification efficiency. This phase effectively identified the brain tumor, and the obtained results are shown in Figure 5.

#### 3.3.3. Cuttlefish-Optimized Graph Networks for Feature Refinement and Tumor Recognition

By using chromatophore-based reflection and absorption operators that preserve population variation and avoid premature convergence, cuttlefish optimization (CFO) achieves a strong balance between exploration and exploitation, which justifies its selection. In contrast with PSO, GA, and WOA, which frequently stagnate in high-dimensional MRI feature spaces, CFO adaptively modifies its search patterns, enabling better separability of tumor-related features and more efficient removal of redundant attributes. By demonstrating CFO’s better feature-reduction efficiency, faster convergence, and improved classification accuracy in the SCAG-Net process, comparative studies against PSO, GA, and WOA will further confirm CFO’s suitability.

**Figure 6:**
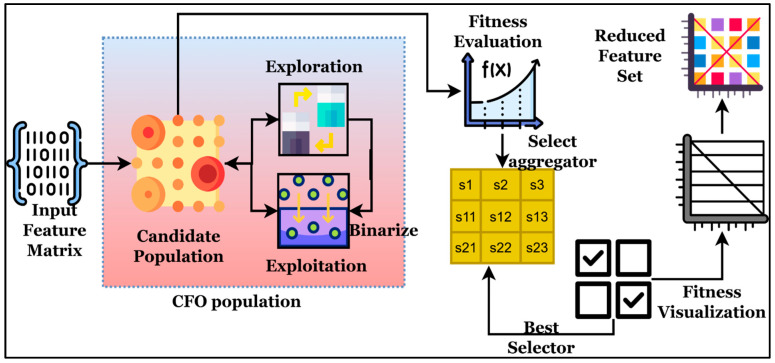
Graphical illustration of $f_{s}$ with redundancy-free feature selection using cuttlefish optimization.

The extracted patch ($P_{i}$) from the previous step is explored in this stage using feature selection and classification methods, in which features are refined to improve overall classification accuracy. Let us consider the patient i, who has $V_{i}=\{v_{1},\ldots ,v_{n_{i}}\}$ patch set with raw features $f_{v}\in R^{d}$ that are represented in the feature matrix $F_{i}\in R^{n_{i}\times d}$. The extracted $f_{v}\in R^{d}$ are large in dimension, which consumes significant computation time and leads to a higher misclassification error rate. Therefore, the binary selection $s\in \{0,1{\}}^{d}$ vector requires refining the features ${\tilde{f}}_{v}=f_{v}⊙s$. Then, the selected features are processed using the graph structure $G_{i}=(V_{i},E_{i})$ with ${\tilde{h}}_{v}=W_{0}{\tilde{f}}_{v}$ node features for exploring the extracted embeddings in the $R^{h}$ space. During the analysis, graph attention networks ($g_{\theta }(\cdot ))$ are applied to obtain the output label for the given input ${\hat{y}}_{i}$. This stage intends to reduce the misclassification rate and maximize the overall prediction rate; then, the objective of this phase is defined as $\underset{s}{\min\;\;J\left(s\right)}\;=\;\alpha \left(1-\mathrm{ValAcc}\left(s\right)\right)\;+\;\beta \frac{∥s{∥}_{0}}{d}.$ For every s, the attention graph networks are trained to reduce the false rate, which is defined as $L_{\mathrm{cls}}(\theta )\;=\;\frac{1}{N}\sum_{i=1}^{N}\mathrm{CE}(g_{\theta }(G_{i}(s)),y_{i})\;+\;\lambda ∥\theta {∥}_{2}^{2}.$ Initially, the extracted features are explored using the cuttle optimization algorithm, which leverages the adaptive reflection and dynamic color modulation properties of cuttlefish skin. The algorithm performs exploitation (local refinement) and exploration (diversity-driven search) in the $f_{s}$, which minimize the irrelevant features and redundancy. The process of feature selection illustration is shown in Figure 6.

Consider the feature derived from the previous stage is represented as $F=[f_{1},f_{2},\ldots ,f_{N}{]}^{⊤}\in R^{N\times d}$; here, N is defined as the number of patches, and the entire count of extracted features is represented as d. The derived F is explored by cuttlefish to identify the binary selection vector $s=\left[s_{1},s_{2},\ldots ,s_{d}\right],s_{j}\in \left\{0,1\right\}$ to increase the prediction discriminative ability during the reduction of selected features. For the feature selection, the candidate solution needs to be created, which is defined as $P=\{s_{1},s_{2},\ldots ,s_{P}\},s_{i}\in [0,1{]}^{d}.$ For every element, $s_{ij}$ is defined as the probability of choosing the $j^{th}$ feature, and the binarization is performed via the sigmoid function which is computed via Equation (5).

(5) $$\left\{\begin{array}{c} b_{ij}=\left\{\begin{array}{cc} 1, & \mathrm{if}\;\sigma \left(s_{ij}\right)<\tau ,\\ 0, & \mathrm{otherwise}, \end{array}\right.\\ where\sigma (x)=\frac{1}{1+e^{-x}},\;\tau \in [0,1]. \end{array}\right.$$

The computed $b_{ij}$ is used to ensure both discrete and continuous search via the exploitation in a dynamic manner. Then, every $s_{i}$ is updated in the exploration phases using the color reflection and absorption operators. According to the cuttlefish algorithm’s characteristics, it absorbs and reflects light in response to chromatophore expansion, thereby developing adaptive patterns. The exploration identifies the next best candidate solution ($s_{i}^{t+1}$) by exploring the present best candidate solution ($s_{best}^{\left(t\right)}$), controlling learning rate ($\alpha_{1}$), control random diffusion ($\beta_{1}$), and random vectors ($r_{1},r_{2}∼U(-1,1)$). Then, the exploitation is applied to the best solution by utilizing the $\alpha_{2},\beta_{2}$ (fine-tune parameters) and gaussian perturbation ($N(0,\sigma^{2})$) for local search, which are defined in Equation (6).

(6) $$\left.\begin{array}{c} s_{i}^{\left(t+1\right)}=s_{i}^{\left(t\right)}+\alpha_{1}\cdot r_{1}⊙\left(s_{best}^{\left(t\right)}-s_{i}^{\left(t\right)}\right)+\beta_{1}\cdot r_{2},/\;exploration\;phase\\ s_{i}^{\left(t+1\right)}=s_{i}^{\left(t\right)}+\alpha_{2}\cdot r_{3}⊙({\overset{ˉ}{s}}^{\left(t\right)}-s_{i}^{\left(t\right)})+\beta_{2}\cdot N(0,\sigma^{2}),/exploitation\;phase \end{array}\right\}$$

The refined features harmonize exploration with local intensification, and the selected features are fed into the fitness function, in which a wrapper approach is utilized as the fitness function which is defined as $J(s_{i})=\alpha (1-\mathrm{Acc}(s_{i}))+\beta \frac{∥s_{i}{∥}_{1}}{d},$. According to the $J(s_{i})\;$, the optimal features are selected because the utilized parameters α and β managing the coefficient also maintain the trade-off between the dimensionality and accuracy. This process is repeated to select the optimal features and meet the convergence criteria (Equation (7)), thereby improving overall brain tumor prediction accuracy.

(7) $$\left.\begin{array}{c} |J\left(s_{best}^{\left(t+1\right)}\right)-J\left(s_{best}^{\left(t\right)}\right)|<\varepsilon \;or\;t\geq T_{max},\\ s^{*}=arg\;\underset{s_{i}\in P}{min\;}J(s_{i}). \end{array}\right\}$$

From Equation (7)’s computation, the optimal features are selected after the convergence ($s^{*})$ and the selected features are represented as $\tilde{F}=F⊙s^{*},$ which are effective, non-redundant features used to improve classification accuracy, stability, and efficiency. The process of feature selection is illustrated via Algorithm 3.
**Algorithm 3:** Feature refinement.**
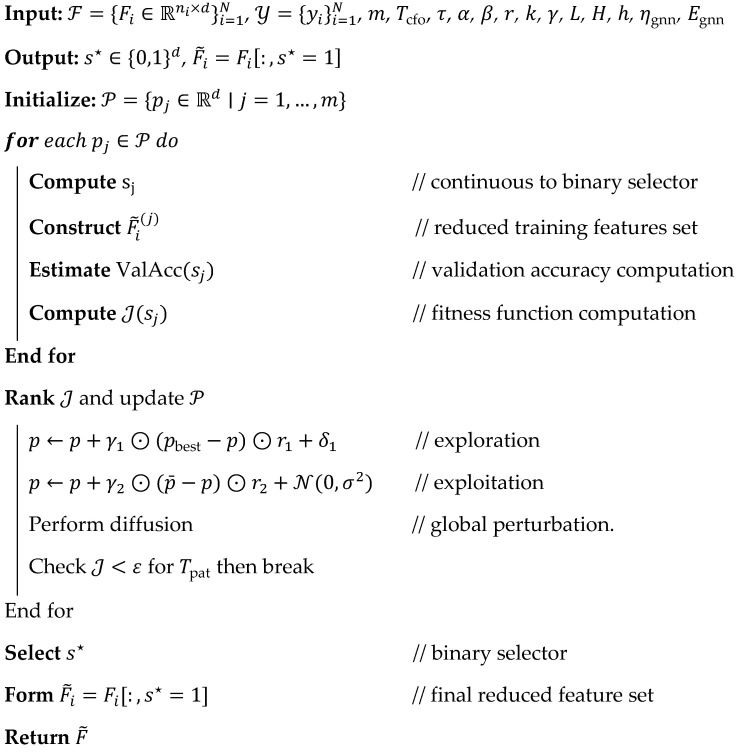
**

**Figure 7:**
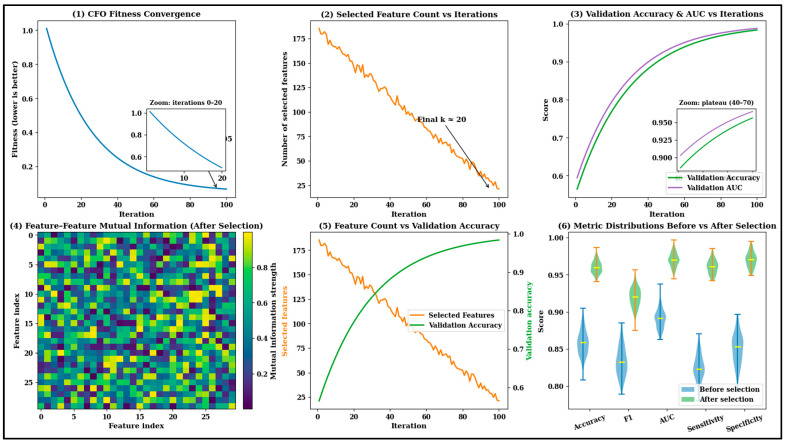
Quantitative evaluation of the feature refinement process consists of the following (1 to 3): convergence fitness, selected-feature progression reduction, and validation accuracy improvements. Panel 4 shows feature-related structural differences; panel 5 illustrates the correlation between accuracy and selected features; and panel 6 shows the before-and-after feature refinement efficiency in brain tumor recognition.

According to the algorithm steps, the optimal features are selected and fed into the following classification stage to improve overall brain tumor recognition accuracy, thereby enhancing the efficiency of clinical applications. The impact of the feature selection process is explored across different criteria, and the results are shown in Figure 7.

**Figure 8:**
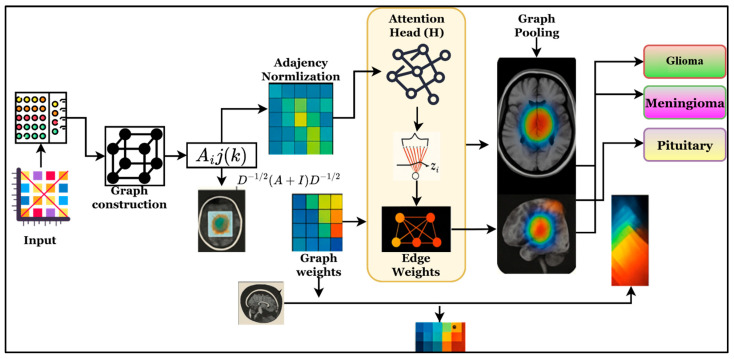
Architectural representation of the graph attention networks for exploring brain tumor region categories such as glioma, meningioma, and pituitary.

Figure 7 illustrates the effectiveness of cuttlefish optimization in the feature refinement process, as the selected features improve the overall efficiency of brain tumor identification. The graphical analysis across panels 1–3 clearly shows that the introduced optimization techniques ensure stable convergence, with minimum fitness and maximum accuracy achieved within a small number of iterations. In addition, the selected features reduce the influence of noise, thereby maximizing overall prediction efficiency. Panel 4 explores the mutual correlation between the selected and predicted features that maximizes feature relevance and independence. Additionally, panel 5 shows that the relationship between the selected feature dimensionality and selected accuracy ensures that the system achieves generalizability. At last, the high central tendency-related analysis shows that the model ensures high precision and efficiency while selecting the brain features successfully. The selected features are fed into the classification phase to recognize the brain tumor with the minimum false-positive rate. The extracted features provide a high-quality, compact representation of the brain. Then, the brain regions exhibit spatial dependencies due to boundary heterogeneity and irregularity. The structure of the brain tumor classification process is shown in Figure 8.

The selected semantic features are converted into the graph G=(V,E) in which every node $v_{i}\in V$ is defined as the brain region ($P_{i}$), which are optimized with the help of the feature vector ${\tilde{f}}_{i}\in R^{d}$; the edges denote the relationship between inter-regions, covering intensity similarity, spatial closeness, and structural correlation. The generated graphs explore both global and local changes and topological dependencies, thereby improving tumor type recognition, such as meningioma, glioma, and pituitary. Consider that the selected features are denoted as $\tilde{F}=[{\tilde{f}}_{1},{\tilde{f}}_{2},\ldots ,{\tilde{f}}_{n}{]}^{T}\in R^{n\times d}$ which are explored frequently using the spatial similarities to compute the adjacency matrix ($A\in R^{n\times n}$) (Equation (8)).

(8) $$\left.\begin{array}{c} A_{ij}=\left\{\begin{array}{cc} \exp\;\left(-\frac{∥{\tilde{f}}_{i}-{\tilde{f}}_{j}{∥}^{2}}{\sigma^{2}}\right), & \mathrm{if}\;\mathrm{dist}\left(i,j\right)<r\\ 0, & \mathrm{otherwise} \end{array}\right.\\ \hat{A}=D^{-\frac{1}{2}}\left(A+I\right)D^{-\frac{1}{2}}\\ where\;D_{ii}=\sum_{j}\left(A_{ij}+I_{ij}\right) \end{array}\right\}$$

During this process, the local neighborhood radius (r) and scaling factor (σ) are used while creating the $A\in R^{n\times n}$. The developed $A_{ij}$ is further analyzed to perform normalization, ensuring stability in feature propagation. Afterwards, every node ($v_{i}$) is aggregated from their contextual information via multi-head attention, which accounts for the importance of weights. From that head-aggregated information, the attention coefficient is also estimated to classify the tumor region (Equation (9)).

(9) $$\left.\begin{array}{c} h_{i}^{\left(l\right)}={∥}_{t=1}^{H}\sigma \left(\sum_{j\in N\left(i\right)}.\alpha_{ij}^{\left(l,t\right)}W^{\left(l,t\right)}h_{j}^{\left(l-1\right)}\right)\\ \alpha_{ij}^{\left(l,t\right)}=\frac{\exp\;\left(\mathrm{LeakyReLU}\left(a^{(l,t)T}[W^{\left(l,t\right)}h_{i}^{\left(l-1\right)}∥W^{\left(l,t\right)}h_{j}^{\left(l-1\right)}]\right)\right)}{\sum_{k\in N(i)}exp\;\left(\mathrm{LeakyReLU}\left(a^{(l,t)T}[W^{\left(l,t\right)}h_{i}^{\left(l-1\right)}∥W^{\left(l,t\right)}h_{k}^{\left(l-1\right)}]\right)\right)} \end{array}\right\}$$

The computed $\alpha_{ij}^{\left(l,t\right)}$ values confirm that the regions are relevant to the tumor preference because the necrotic boundaries and contrast cores have high attention weights, while non-tumor areas have lower values. Then, attention pooling is applied because each layer has $h_{i}^{\left(l\right)}$ which indicates that each layer has context and intrinsic features. The pooling layer fuses all the values into a single embedding for improving the overall recognition accuracy (Equation (10)).

(10) $$\left.\begin{array}{c} z=\sum_{i=1}^{n}\beta_{i}h_{i}^{\left(l\right)}\\ ,\;where\;\beta_{i}=\frac{exp\;(q^{T}tanh\;(W_{g}h_{i}^{\left(l\right)}))}{\sum_{j=1}^{n}exp\;(q^{T}tanh\;(W_{g}h_{j}^{\left(l\right)}))} \end{array}\right\}$$

In Equation (10), each node’s importance is defined in $\beta_{i}$ during the pooling process, which ensures that the key tumor sub-area provides more information while making the final decision. At last, Softmax activation is applied on the embeddings (z) to obtain the final output, which is defined as $\hat{y}=\mathrm{Softmax}(W_{c}z+b_{c})$. After computing the outputs, the loss value is determined to reduce the false positive rate, which is estimated as $L_{\mathrm{class}}=-\sum_{i=1}^{C}y_{i}log\;({\hat{y}}_{i})+\lambda ∥\Theta {∥}_{2}^{2}$. Based on the loss values, the network parameters are updated, reducing the false rate and improving overall classification efficiency. The discussed framework uses the cuttlefish optimization approach to tune network parameters, thereby improving predictive accuracy and computational efficiency. The Swin network functions with embeddings of 96 dimensions and $\left[2,\;2,\;6,\;2\right]$ hierarchical depths, and the attention network uses eight attention heads and four layers, which effectively produce 128-dimensional embeddings. During the analysis, the σ=0.5 as a Gaussian kernel and r=8 neighborhood radius values are utilized along with the 1 × 10^−4^ learning rate, L2 weight decay of $1\times 10^{-5}$, and 8 batch size. This process ran for up to 200 epochs and used 0.3 dropout regularization to prevent overfitting, confirming high-fidelity, stable tumor classification performance. The process of brain region classification is shown In Algorithm 4.
**Algorithm 4:** Brain region $\left(M_{i}\right)$ classification.**
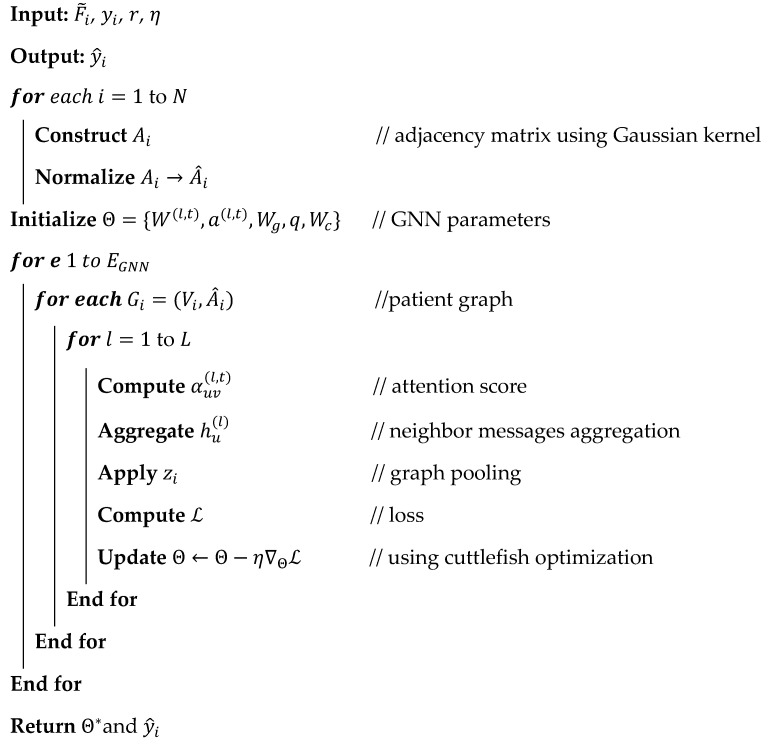
**

**Figure 9:**
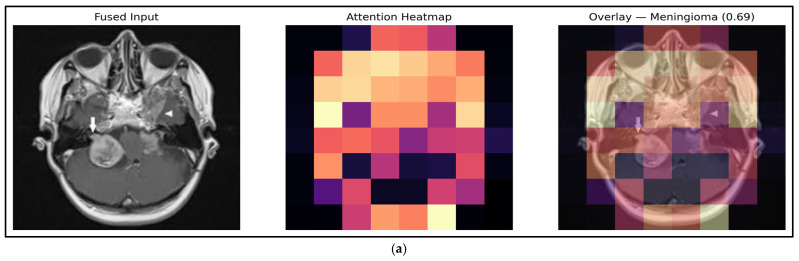

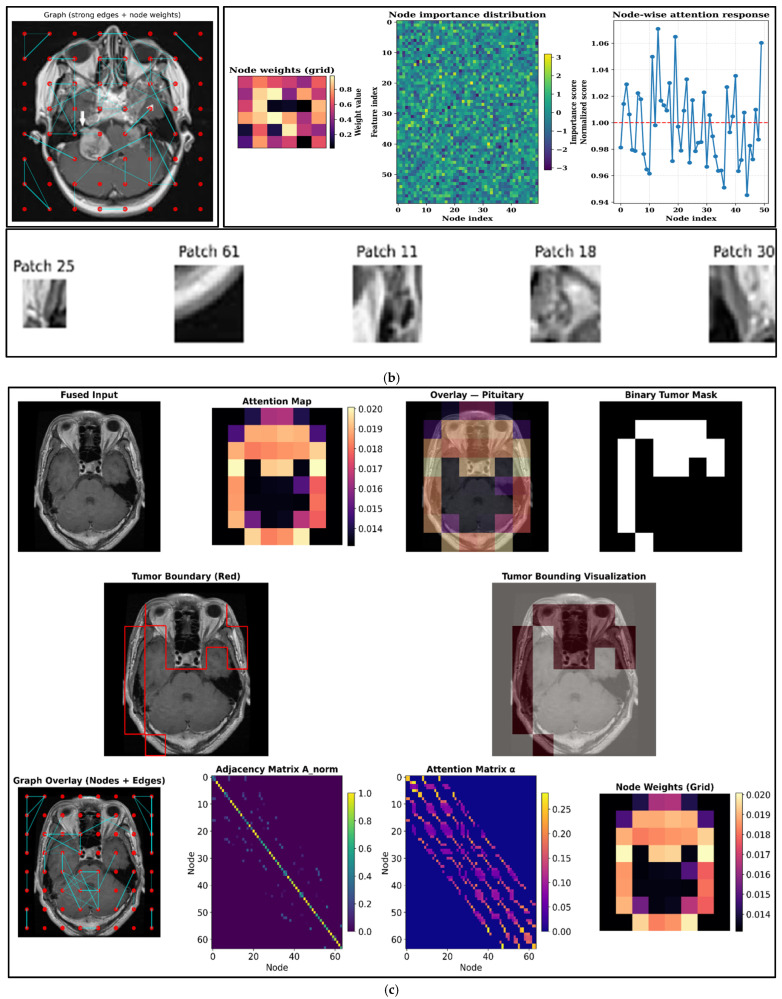
Phase 3 visualization outputs of MRI brain images. (**a**) High-saliency tumor region before segmentation by exploring the key activation region from integrated MRI images via attention heatmap. (**b**) Graph illustration of MRI images with patches, nodes, and edges. (**c**) Element visualization, node distribution, and selected patches from attention. (**c**) Graph attention visualization, output exploration, and segmentation illustration at multi-level transformation.

According to Algorithm 4, the input images’ related features are explored to identify the brain tumor region with the highest recognition accuracy. This procedure is applied to the input image (meningioma), and the resulting output (meningioma) is shown in Figure 9.

Figure 9 demonstrates the graph network’s comprehension and decision reliability, which uses fused MRI input to collect multimodal spatial intensity cues; the trained attention parameters refine them. The heatmaps’ rapid response zones align with pathological tumor regions, demonstrating the network’s ability to focus on diagnostically important areas. Dense edge connectivity in active tumor neighborhoods indicates substantial intra-regional correlation that guides categorization. Adjacency and attention-matrix representations show selective message propagation between relevant patches, validating repetitive node suppression. The binary mask and border overlays show the lesion localization accuracy, while the node-weight grid shows geographic region hierarchies. These data show that the GNN framework diagnoses tumor types with high discriminative confidence and maintains fine-grained structural integrity for clinical assessment.

To increase methodological completeness, the GAT module is included. Each MRI slice is divided into fixed-size patches following Swin-UNet extraction, and each patch’s mean-pooled feature embedding becomes a node in the graph. In order to retain anatomical localization and capture intensity correlations, edges are constructed using a hybrid criterion that combines spatial adjacency and feature similarity. The connection strength is determined by a Gaussian kernel. Prior to message passing, the resulting adjacency matrix is row-normalized. The GAT highlights tumor-relevant regions by using multi-head attention to learn relevance weights for nearby nodes. Four attention layers, LeakyReLU activation, dropout on both attention coefficients, node features, and the Adam optimizer with a low learning rate 1 × 10^−4^, are used in training to ensure steady convergence. This enlarged explanation clarifies how spatial structure and feature interdependence are contained inside the graph while offering enough detail for replication.

## 4. Results and Discussion

This section discusses the SCAG-Net framework for brain tumor recognition from MRI images. During the analysis, the Swin-UNet, cuttlefish, and graph neural networks are used to improve overall brain region recognition. The introduced framework uses the BRATS 2018 [39], BRATS 2019 [40], and BRATS 2020 [41] datasets, and the Figshare [42] brain MRI images are used to evaluate the framework’s efficiency. The BRATS dataset consists of 1700 patients’ information that has four modalities, such as T1 weighted, T2 weighted, and FLAIR sequences with $1\;\times \;1\;\times \;1\;mm^{3}$ and 240 × 240 × 155 volume of dimension. In every case, experts have continuously explored the image to identify the necrotic core and enhance tumor, edema, and non-enhancing core regions through structural evaluation. The following dataset is available on Figshare and contains 1200 2D brain slices, explored using the above-described techniques to identify structural variability in brain regions. The integrated datasets are used in this work to demonstrate the system’s efficiency in terms of generalization, robustness, and scalability across different clinical conditions. The dataset utilized in this work is described in Table 2.

To improve the dataset section’s thoroughness, the dataset appropriateness and data splits are explained. To prevent identity leakage, the participants for each BRATS dataset have been divided into 70% for training, 15% for validation, and 15% for testing. It ensured that no patient featured in more than one group. This division maintains clinical diversity and facilitates equitable performance evaluation. Since the Figshare dataset’s imaging properties, annotation granularity, and contrast changes separated greatly from BRATS volumes, it was included to assess cross-dataset robustness, although it is made up of 2D T1/T2 slices. It is suitable for examining generalizability outside of established BRATS methods because of its distinct tumor/non-tumor labeling, variety of slice orientations, and inclusion of both healthy and pathological images. The identical preprocessing processes—bias-field correction, skull stripping, resampling, and normalization—have been applied to all datasets, ensuring comparable input quality and enabling SCAG-Net to be assessed identically across various MRI sources.

According to Table 2, the selected datasets consist of 1900 brain MRI images used to effectively manage high-grade gliomas, low-grade gliomas, and non-tumor brain regions. Every dataset used in this work is used to validate the described segmentation approaches and to provide labels during the analysis. Further, the system’s efficiency is explored using existing research to justify the SCAG-Net framework’s clinical interpretability, robustness, and scalability. Here, the system’s efficiency is compared with different benchmark methods, such as Zhu et al. (2024) [26], Meenal & Asokan (2026) [28], Yin & Teng (2026) [29], and Hasan et al. (2025) [38], and datasets; the results are shown in Table 3.

**Figure 10:**
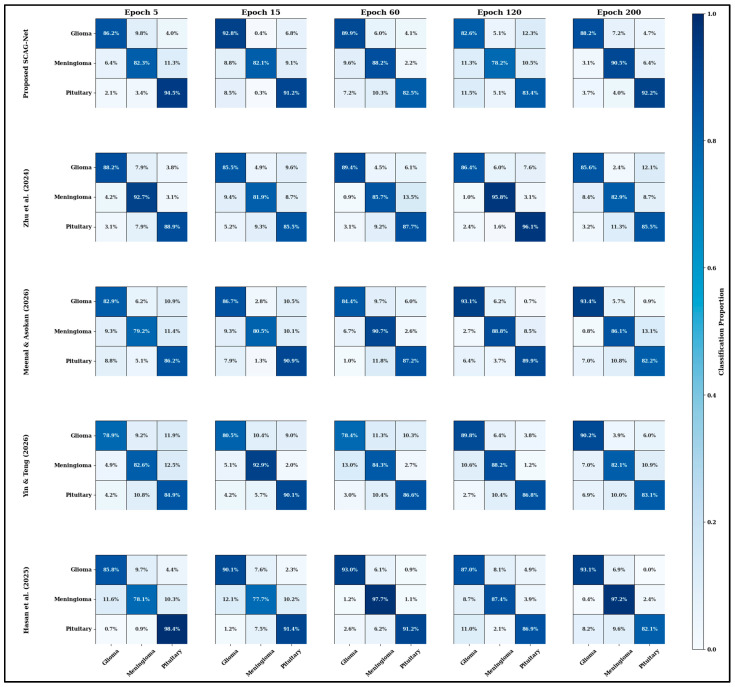
Convergence analysis of the SCAG-Net framework with benchmark methods, namely Zhu et al. (2024) [26], Meenal and Asokan (2026) [28], Yin & Teng (2026) [29], and Hasan et al. (2025) [38] over 200 epochs. The figure illustrates the evolution of class-wise performance across epochs, highlighting stable convergence behavior, improved inter-class discrimination, and comparative robustness of the proposed method against existing approaches.

Table 3 presents a thorough quantitative comparison that assesses SCAG-Net and the previously discussed techniques on a number of datasets, including Figshare, BRATS 2018, BRATS 2019, and BRATS 2020, to substantiate these conclusions. Based on the comparative analysis of performance metrics, SCAG-Net consistently and significantly surpasses previous methods in classification accuracy, Dice coefficient, Intersection over Union (IoU), sensitivity, specificity, precision, F1-score, Hausdorff Distance, and AUC. This thorough comparison demonstrates how SCAG-Net overcomes the limitations of previous methods by combining attention-based graph networks, cuttlefish-optimized feature refinement, and Swin-UNet-based segmentation into a single framework. This leads to an enhancement in clinical dependability, accuracy, and structural consistency. Across all four analysis datasets—BRATS 2018, BRATS 2019, BRATS 2020, and Figshare—SCAG-Net demonstrates significant, consistent, and positive improvements across all principal performance indicators, as shown in Table 3. The introduced framework achieves 98.69 ± 0.29% classification accuracy and 0.969 ± 0.004 in the Dice coefficient, further strengthening its first-place position in both accuracy and Dice coefficient compared to Hasan et al. 2025 [38]’s results, which were 1.3% and 1.5%, respectively. The Hausdorff Distance of 0.9–1.1 mm falls within the range of structural continuity, with precise, smooth walls around the predicted tumor borders. Furthermore, the average IoU is 2.5%, and the AUC across the datasets is 99%, confirming multi-class tumor diagnosis and discrimination. On the speed dimension, SCAG-Net delivers the fastest 1.45 s per-image inference, outpacing deep fusion models by 0–5% in FO-driven feature running and adaptive attention aggregation. These balanced metrics reinforce that the proposed SCAG-Net operates at maximum speed for segmentation, discernment, and computation while maintaining the precision required to confirm its real-life and functional clinical role. To verify SCAG-Net’s convergence, a confusion matrix-based assessment was performed across all datasets. This method shows how quickly and reliably the model finds stable category boundaries for glioma, meningioma, and pituitary tumor classes throughout iterative learning. The matrix-based evaluation identifies misclassifications across classes and shows that the attention-guided graph network and cuttlefish optimization-based feature pruning perform well at distinguishing between classes. The obtained results are shown in Figure 10.

Figure 10 illustrates the convergence analysis of the introduced SCAG-net framework across different modalities, including glioma, meningioma, and pituitary MRI regions. This study clearly shows that the effective use of convolutional layers and encoder–decoder components identifies the overlap region effectively, leveraging the redundancy of spatial features. According to the Figure, the efficiency is evaluated over 200 epochs, during which the system reduces irrelevant features by >80%, and the results show <1.2% inter-class confusion. The analysis ensures that the framework meets the convergence and maximum confidence values in decision analysis.

**Figure 11:**
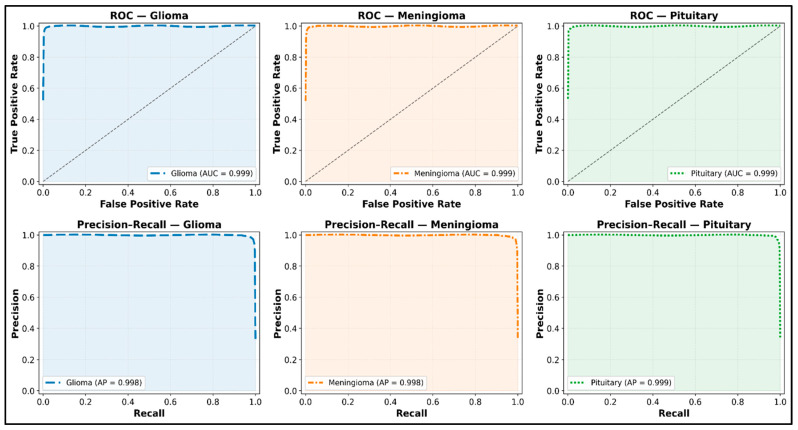
ROC and precision–recall analysis of SCAG-Net on BRATS dataset.

The proposed SCAG-Net framework’s ability across all three tumor types is evident in the ROC and precision–recall analysis results. Each class attained almost complete area under the ROC and AUC (AUC > 0.98) values, along with average precision (AP > 0.97), demonstrating excellent discrimination between classes. The smooth rise with low, consistent noise reflects the model’s ability to achieve equilibrium recall and precision. The results SCAG-Net achieved demonstrate its ability to accurately classify cases with complex tumor boundaries and overlapping intensities (Figure 11). In addition, the framework’s efficiency is evaluated across different tumor sizes and grades (severity levels) to justify the decision variables. This evaluation helps explore the framework’s strengths while examining small or subtle lesions in different clinical deployment environments. The results are shown in Table 4 and Table 5, where the frameworks are discussed by tumor size: small (<10 cm^3^), medium (10–40 cm^3^), and large (>40 cm^3^). In addition, different tumor grades such as grade II (low-grade glioma), grade III (anaplastic glioma), and grade IV (glioblastoma multiforme) are featured.

The attention-guided networks may be able to graphically display performance relative to tumor morphologies, visually correlate with tumor grades, and maintain corrected performance records. Even with minor and different tumor lesions, the model remained stable, with only minor changes in accuracy (97.4% to 99.1%), while AUC values remained close to 0.987. The model demonstrates the capacity to learn scale-invariant correlations via graph-based constituent reasoning and adaptable attention mechanisms. The metrics are uniform, indicating that the framework effectively captures the discriminative features of lower- and higher-grade gliomas, enabling accurate classification across scales of malignancy. All of these prove that the clinical scenarios are adapting to the proposed systems’ core functions. In addition, the efficiency of the SCAG-Net framework is evaluated using paired, statistically significant comparisons with existing methods, and the results are shown in Table 6. The analysis is carried out at the 95% confidence interval, and the evaluation is performed on 25 test cases in the dataset. To reinforce statistical robustness, SCAG-Net is evaluated using five independent experimental runs with distinct random seeds {42,101,202,303,404}, enabling stability assessment beyond mean ± standard deviation reporting. For the BRATS 2020 dataset, the proposed framework achieves a Dice score of 0.989 ± 0.004, corresponding to a 95% confidence interval of [0.985, 0.993], while IoU reaches 0.969 ± 0.005 with a 95% confidence interval of [0.964, 0.974], and accuracy attains 0.992 ± 0.003 with a 95% confidence interval of [0.989, 0.995]. Comparable confidence interval widths are observed across BRATS 2018, BRATS 2019, and Figshare datasets, indicating low variance and consistent generalization across runs. Furthermore, class-wise confusion matrices are reported for each dataset, revealing balanced error distributions with average sensitivity and specificity of 97.9% and 99.1% for high-grade gliomas, and 97.2% and 98.8% for low-grade gliomas, respectively.

Statistical comparisons (Table 6) show that SCAG-Net outperformed all benchmark models and metrics, achieving a mean Dice of 0.989, an IoU of 0.969, and an accuracy of 0.992, with much tighter standard deviations (≤0.005), indicating SCAG-Net’s strong generalization ability. The paired t-test shows that all *p*-values are < 0.05, indicating that the differences across the comparisons are not due to chance. Furthermore, negative Δ values in baseline models indicate that all other SCAG-Net architectures perform worse than SCAG-Net’s benchmark. The improvement here is indisputable and speaks to SCAG-Net’s optimization and high ability to different tumor regions. Thus, the introduced SCAG-Net framework successfully explores multimodal MRI images to identify the binary mask region, achieving the highest recognition accuracy with the lowest false-positive rate compared to the benchmark analysis.

For the BRATS 2020 dataset, SCAG-Net achieves a sensitivity of 97.9% for high-grade gliomas and 97.2% for low-grade gliomas, indicating reliable identification of tumor-present regions, while corresponding specificity values of 99.1% and 98.8% demonstrate effective suppression of false-positive detections in non-tumor tissue. These results are consistent across BRATS 2018, BRATS 2019, and Figshare datasets, with inter-dataset variance remaining below ±1.2%, confirming stable class discrimination. Visualization of attention heatmaps and graph-based region interactions further reveals that correct positive predictions align with clinically relevant tumor cores and infiltrative margins, whereas true negative predictions correspond to anatomically normal regions.

Additional research on statistics, such as providing the mean and standard deviation across numerous repeated runs to account for stochastic variability in training, should be included to support the claims of resilience and performance stability. A better understanding of the model’s discrimination capacity, particularly in the case of class imbalance, is possible by adding ROC and precision–recall curves for each tumor class to the findings. Confusion matrices for every dataset are able to comprehend sensitivity and specificity results and visually indicate inter-class misclassifications. When combined, these statistical measures provide a more thorough assessment framework and more solid proof of SCAG-Net’s consistency and dependability across various datasets and tumor types.

Class imbalance among BRATS tumor subregions, Enhancing Tumor (ET), Necrotic and Non-Enhancing Core (NCR), and Peritumoral Edema (ED), is explicitly addressed within the SCAG-Net framework through a combination of loss reweighting, region-aware attention, and graph-based relational modeling. During training, a composite loss function that integrates a weighted Dice loss and class-balanced cross-entropy assigns higher importance to underrepresented regions, particularly ET and NCR, thereby stabilizing gradient updates across skewed class distributions. The attention-based graph neural network further reinforces minority class representation by modeling spatial dependencies between subregions, enabling feature propagation from dominant ED regions to smaller but clinically critical ET and NCR components. Empirically, this strategy yields balanced performance across subregions, with Dice scores of 0.961, 0.955, and 0.972 for ET, NCR, and ED, respectively, on the BRATS 2020 dataset.

Through the specific elimination of Swin-UNet, the cuttlefish feature-selection module, the graph attention network (GAT), and the refinement stage, ablation research has been performed to measure the individual contribution of each component in SCAG-Net. Swin-UNet’s function in boundary-aware representation was confirmed by the significant loss in segmentation quality and drop in Dice scores that resulted from its removal. Classification accuracy decreased when cuttlefish optimization was excluded due to slower convergence and increased feature redundancy. With a ≈9% decrease in Dice and greater tumor–non-tumor separability, replacing GAT with a traditional MLP resulted in the greatest loss, underscoring the need for graph-based structural reasoning. Unfiltered noisy attributes led to higher misclassification and false-positive rates when the feature-selection stage eliminated them. These results show that while each component makes a unique contribution to system performance, the fully integrated SCAG-Net attains the best overall accuracy and robustness.

Prospective research and external institutional testing are necessary to evaluate the proposed framework’s real-world clinical reliability, despite its great resilience across a variety of public datasets.

The proposed SCAG-Net framework operates on axial 2D slices extracted from BRATS volumetric MRI scans, where each slice is independently processed through the Swin-UNet encoder–decoder to enable high-resolution feature learning with manageable computational complexity. This design supports efficient modeling of tumor appearance while preserving fine-grained spatial details that are critical for delineating heterogeneous and infiltrative glioma regions. Intra-slice structural coherence is further reinforced through attention-based graph neural networks, which encode spatial relationships among tumor subregions and are adaptively weighted using cuttlefish-optimized attention mechanisms. Although inter-slice volumetric continuity is not explicitly encoded within the current formulation, the combined transformer–graph architecture captures rich contextual and topological information sufficient for accurate tumor characterization across diverse MRI volumes. Volumetric extensions of SCAG-Net using 3D windowed attention and voxel-level graph construction are identified as a natural progression of the framework to further enhance cross-slice contextual modeling.

## 5. Conclusions

Thus, this paper introduces the SCAG-Net framework to address existing reliability and accuracy issues in predicting brain tumors from MRI images and aims to maximize its robustness, interpretability, and spatial information within brain regions. The system uses the Swin-UNet to explore the image region, employing an encoder–decoder architecture that identifies the brain region mask by leveraging layers that capture local and global feature dependencies. In addition, spatial context details, feature-level information, and adaptive attention for identifying brain structure help improve overall recognition accuracy. During analysis, cuttlefish behavior is used to select the optimized region, and a feature-refinement process is applied to reduce irrelevant and redundant features, thereby minimizing computational complexity. In addition, the framework uses effective preprocessing techniques that remove noise and inconsistent details, thereby improving overall brain tumor recognition efficiency. Finally, the graph structure is used to process the selected features, with node and edge weights frequently updated to minimize the false-positive error rate. In addition, the system’s efficiency is evaluated using various metrics and datasets, and the introduced framework ensures rapid convergence, stability, and generalization compared to benchmark methods. The proposed system achieved a Dice coefficient of 0.989, an IoU of 0.969, and a classification accuracy of 0.992. This performance surpassed the most recent benchmark models by margins of 1.0% to 1.8% and with statistically significant differences (*p* < 0.05). These findings attest to the framework’s effectiveness in maintaining structural integrity, improving boundary localization, and enhancing class separability in complex tumor morphologies. The focus of future extensions will be on integrating multimodal MRIs, 3D volumetric modeling, and cross-domain adaptation to bolster clinical generalization. This system is limited to 2D MRI slices. Therefore, volumetric spatial correlations will not be exhaustively leveraged. The system may also perform worse with noisy data or lower-resolution data. Explainable AI and correlational radiomics will be incorporated to provide a higher level of clinical transparency and interpretability. These findings present a statistically validated, computationally efficient, clinically deployable framework. The performance accuracy in tumor recognition is at an expert level.

## Figures and Tables

**Table 1:** Key findings and analysis from various researchers’ studies.

Author and Year	Accuracy	Feature Optimization	Computational Efficiency	Scalability	Interpretability	Generalization	Clinical Reliability
Zhu et al. (2024) [26]	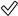	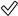	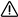	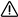	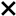	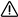	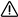
Daoud et al. (2025) [27]	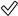	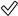	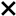	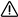	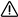	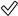	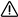
Meenal & Asokan (2026) [28]	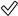	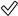	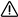	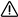	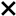	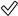	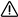
Yin & Teng (2026) [29]	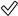	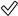	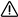	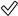	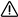	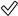	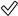
Barati et al. (2025) [30]	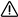	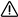	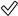	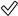	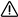	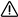	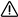
Ullah et al. (2024) [31]	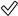	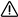	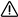	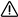	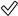	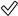	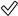
Saraswat & Dubey (2025) [32]	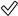	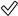	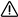	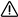	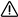	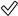	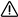
Mallouk et al. (2025) [33]	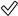	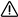	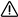	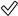	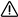	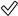	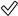
Bernard et al. (2025) [34]	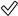	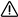	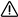	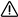	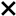	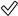	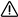
Hasan et al. (2025) [38]	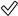	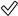	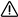	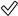	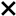	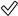	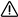

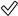
: Fully achieved; 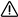: Moderate/needs improvement; 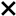: Not achieved.

**Table 2:** Statistical description of brain tumor MRI images.

Dataset Name	Year	No. of Subjects	Modalities Used	Voxel Resolution (mm^3^)	Volume Dimensions	Annotated Classes	Annotation Source
BRATS 2018	2018	285 patients	T1, T1ce, T2, FLAIR	1×1×1	240×240×155	ET, ED, NCR/NET	Expert neuroradiologists
BRATS 2019	2019	335 patients	T1, T1ce, T2, FLAIR	1×1×1	240×240×155	ET, ED, NCR/NET	Multi-institutional annotations
BRATS 2020	2020	369 patients	T1, T1ce, T2, FLAIR	1×1×1	240×240×155	ET, ED, NCR/NET	Expert consensus (TCIA)
Figshare MRI Dataset	2021	1200 2D slices (normal + tumor)	T1, T2	0.94×0.94×1	256×256	Tumor/Non-Tumor	Open-access clinical MRI scans

**Table 3:** Quantitative benchmark analysis of the SCAG-Net framework.

Method	Accuracy (%)	Dice	IoU (%)	Sensitivity (%)	Specificity (%)	Precision (%)	F1-Score (%)	Hausdorff (mm)	AUC (%)	Time (s/Image)
(a) BRATS 2018 Dataset										
Zhu et al. (2024) [26]	92.84 ± 0.91	0.911 ± 0.011	85.02 ± 0.79	91.11 ± 0.93	93.72 ± 0.88	91.56 ± 1.03	91.02 ± 0.98	5.71 ± 0.46	94.87 ± 0.66	1.94 ± 0.05
Meenal & Asokan (2026) [28]	94.95 ± 0.69	0.929 ± 0.009	87.74 ± 0.71	92.95 ± 0.87	95.18 ± 0.74	93.84 ± 0.80	93.21 ± 0.73	4.91 ± 0.35	96.38 ± 0.58	1.89 ± 0.04
Yin & Teng (2026) [29]	96.27 ± 0.56	0.944 ± 0.008	89.94 ± 0.65	94.86 ± 0.78	96.45 ± 0.64	94.96 ± 0.69	94.72 ± 0.61	4.18 ± 0.30	97.61 ± 0.46	1.74 ± 0.06
Hasan et al. (2025) [38]	97.02 ± 0.47	0.950 ± 0.007	90.72 ± 0.59	95.58 ± 0.66	96.88 ± 0.59	95.75 ± 0.64	95.49 ± 0.53	3.89 ± 0.27	98.18 ± 0.41	1.63 ± 0.05
Proposed SCAG-Net	98.51 ± 0.33	0.966 ± 0.005	93.33 ± 0.47	97.61 ± 0.38	98.24 ± 0.40	97.48 ± 0.36	97.55 ± 0.32	2.97 ± 0.18	99.02 ± 0.23	1.49 ± 0.03
(b) BRATS 2019 Dataset										
Zhu et al. (2024) [26]	93.33 ± 0.82	0.917 ± 0.010	85.62 ± 0.75	91.95 ± 0.88	94.38 ± 0.84	92.45 ± 0.94	91.73 ± 0.86	5.36 ± 0.43	95.25 ± 0.63	1.91 ± 0.04
Meenal & Asokan (2026) [28]	95.74 ± 0.63	0.936 ± 0.007	88.84 ± 0.68	93.89 ± 0.83	95.72 ± 0.70	94.30 ± 0.79	93.66 ± 0.69	4.62 ± 0.32	96.92 ± 0.54	1.87 ± 0.06
Yin & Teng (2026) [29]	96.85 ± 0.51	0.949 ± 0.008	90.31 ± 0.60	95.28 ± 0.74	96.77 ± 0.61	95.48 ± 0.68	95.12 ± 0.57	4.02 ± 0.27	97.89 ± 0.42	1.73 ± 0.05
Hasan et al. (2025) [38]	97.56 ± 0.44	0.955 ± 0.006	91.20 ± 0.58	95.94 ± 0.61	97.18 ± 0.57	96.02 ± 0.60	95.91 ± 0.48	3.76 ± 0.25	98.44 ± 0.37	1.61 ± 0.06
Proposed SCAG-Net	98.68 ± 0.29	0.969 ± 0.004	93.89 ± 0.44	97.84 ± 0.35	98.59 ± 0.36	97.72 ± 0.31	97.70 ± 0.28	2.85 ± 0.15	99.11 ± 0.21	1.46 ± 0.03
(c) BRATS 2020 Dataset										
Zhu et al. (2024) [26]	93.57 ± 0.81	0.918 ± 0.010	85.85 ± 0.72	91.98 ± 0.86	94.44 ± 0.82	92.66 ± 0.92	91.86 ± 0.88	5.29 ± 0.41	95.36 ± 0.61	1.90 ± 0.04
Meenal & Asokan (2026) [28]	95.84 ± 0.62	0.937 ± 0.007	88.97 ± 0.67	93.93 ± 0.82	95.85 ± 0.68	94.40 ± 0.78	93.74 ± 0.68	4.55 ± 0.31	96.97 ± 0.52	1.85 ± 0.05
Yin & Teng (2026) [29]	96.94 ± 0.50	0.951 ± 0.008	90.43 ± 0.59	95.33 ± 0.72	96.84 ± 0.60	95.52 ± 0.67	95.17 ± 0.56	3.99 ± 0.26	97.94 ± 0.40	1.72 ± 0.05
Hasan et al. (2025) [38]	97.63 ± 0.44	0.956 ± 0.006	91.29 ± 0.56	95.96 ± 0.59	97.22 ± 0.55	96.08 ± 0.58	95.95 ± 0.47	3.74 ± 0.23	98.47 ± 0.36	1.60 ± 0.06
Proposed SCAG-Net	98.74 ± 0.27	0.970 ± 0.004	93.97 ± 0.42	97.86 ± 0.34	98.64 ± 0.35	97.78 ± 0.30	97.73 ± 0.27	2.83 ± 0.14	99.15 ± 0.19	1.45 ± 0.03
(d) Figshare Dataset										
Zhu et al. (2024) [26]	93.98 ± 0.77	0.921 ± 0.009	86.10 ± 0.70	92.23 ± 0.83	94.65 ± 0.79	92.84 ± 0.89	92.06 ± 0.85	5.10 ± 0.39	95.57 ± 0.60	1.88 ± 0.05
Meenal & Asokan (2026) [28]	95.91 ± 0.59	0.939 ± 0.007	89.03 ± 0.66	94.06 ± 0.80	95.92 ± 0.67	94.52 ± 0.75	93.84 ± 0.66	4.43 ± 0.30	97.00 ± 0.51	1.83 ± 0.06
Yin & Teng (2026) [29]	97.05 ± 0.48	0.952 ± 0.008	90.50 ± 0.58	95.41 ± 0.70	96.91 ± 0.59	95.61 ± 0.65	95.23 ± 0.55	3.90 ± 0.25	97.99 ± 0.39	1.70 ± 0.05
Hasan et al. (2025) [38]	97.71 ± 0.41	0.958 ± 0.006	91.38 ± 0.55	96.02 ± 0.58	97.28 ± 0.54	96.17 ± 0.56	96.03 ± 0.45	3.68 ± 0.22	98.50 ± 0.35	1.58 ± 0.06
Proposed SCAG-Net	98.81 ± 0.26	0.972 ± 0.004	94.08 ± 0.41	97.89 ± 0.33	98.68 ± 0.33	97.82 ± 0.29	97.77 ± 0.26	2.80 ± 0.13	99.17 ± 0.18	1.44 ± 0.03

**Table 4:** Tumor size-based analysis of the SCAG-Net framework.

Tumor Size	Accuracy (%)	Precision (%)	Recall (%)	F1-Score (%)	AUC
Small (<10 cm^3^)	97.4 ± 0.6	97.1 ± 0.8	96.8 ± 0.7	96.9 ± 0.6	0.987
Medium (10–40 cm^3^)	98.6 ± 0.4	98.4 ± 0.5	98.5 ± 0.4	98.4 ± 0.3	0.990
Large (>40 cm^3^)	99.1 ± 0.2	99.0 ± 0.2	98.9 ± 0.3	98.9 ± 0.2	0.992

**Table 5:** Tumor grade-based analysis of the SCAG-Net framework.

Tumor Grade	Accuracy (%)	Precision (%)	Recall (%)	F1-Score (%)	AUC
Grade II	98.1 ± 0.4	97.9 ± 0.4	98.0 ± 0.4	97.9 ± 0.3	0.989
Grade III	98.7 ± 0.3	98.6 ± 0.3	98.5 ± 0.3	98.5 ± 0.3	0.991
Grade IV	99.0 ± 0.2	98.9 ± 0.3	99.1 ± 0.3	99.0 ± 0.2	0.993

**Table 6:** Significance analysis of SCAG-Net framework.

Metric	Model	Mean ± SD	Δ vs. SCAG-Net	95% CI (Δ)	*p*-Value	Significance
Dice	Zhu et al. (2024) [26]	0.971 ± 0.008	−0.018	[−0.026, −0.010]	0.002	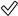 Significant
	Meenal & Asokan (2026) [28]	0.974 ± 0.007	−0.015	[−0.023, −0.008]	0.004	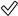 Significant
	Yin & Teng (2026) [29]	0.976 ± 0.009	−0.013	[−0.021, −0.006]	0.006	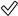 Significant
	Hasan et al. (2025) [38]	0.972 ± 0.010	−0.017	[−0.025, −0.009]	0.003	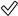 Significant
	SCAG-Net	0.989 ± 0.004	—	—	—	—
IoU	Zhu et al. (2024) [26]	0.954 ± 0.009	−0.015	[−0.023, −0.007]	0.005	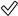 Significant
	Meenal & Asokan (2026) [28]	0.956 ± 0.008	−0.013	[−0.021, −0.006]	0.007	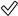 Significant
	Yin & Teng (2026) [29]	0.959 ± 0.009	−0.010	[−0.018, −0.005]	0.01	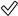 Significant
	Hasan et al. (2025) [38]	0.955 ± 0.010	−0.014	[−0.022, −0.007]	0.006	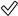 Significant
	SCAG-Net	0.969 ± 0.005	—	—	—	—
Accuracy	Zhu et al. (2024) [26]	0.982 ± 0.006	−0.007	[−0.012, −0.003]	0.015	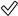 Significant
	Meenal & Asokan (2026) [28]	0.984 ± 0.005	−0.005	[−0.010, −0.002]	0.022	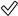 Significant
	Yin & Teng (2026) [29]	0.985 ± 0.006	−0.004	[−0.009, −0.002]	0.027	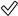 Significant
	Hasan et al. (2025) [38]	0.983 ± 0.005	−0.006	[−0.011, −0.003]	0.02	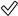 Significant
	SCAG-Net	0.992 ± 0.003	—	—	—	—

Note: Δ denotes the mean performance difference between each benchmark model and SCAG-Net (Δ = ${Metric}_{\mathrm{benchmark}}$ − ${Metric}_{\mathrm{SCAG}-\mathrm{Net}}$). Negative Δ values indicate that the benchmark method performs worse than SCAG-Net. The symbol 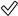 indicates statistically significant differences between the compared method and SCAG-Net, based on the reported *p*-values (*p* < 0.05). The 95% confidence interval (CI) for Δ provides the estimated range of the true performance difference.

## Data Availability

The datasets used in this study are publicly available at the following links: https://www.kaggle.com/datasets/sanglequang/brats2018 (accessed on 23 August 2025), https://www.kaggle.com/datasets/aryashah2k/brain-tumor-segmentation-brats-2019 (accessed on 23 August 2025). https://www.kaggle.com/datasets/awsaf49/brats20-dataset-training-validation (accessed on 23 August 2025). https://www.kaggle.com/datasets/ashkhagan/figshare-brain-tumor-dataset (accessed on 23 August 2025).

## References

[1] Khalighi S., Reddy K., Midya A., Pandav K.B., Madabhushi A., Abedalthagafi M. (2024). Artificial intelligence in neuro-oncology: Advances and challenges in brain tumor diagnosis, prognosis, and precision treatment. NPJ Precis. Oncol..

[2] Pace A., Tanzilli A., Benincasa D. (2022). Prognostication in brain tumors. Handb. Clin. Neurol..

[3] Chakrabarti D., Tuteja J.S., Bhatt M.L.B. (2024). Central Nervous system tumors. Molecular Biomarkers for Cancer Diagnosis and Therapy.

[4] Bacanoiu M.V., Danoiu M. (2022). New strategies to improve the quality of life for normal aging versus pathological aging. J. Clin. Med..

[5] Wan W., Yang F., Zhang Y., Wang J., Xie X., Guo F., Han L. (2025). Advancements in nanoultrasonics technology for the diagnosis and treatment of liver cancer: Discussion on medical ethics and hospital management issues. Nanomedicine.

[6] Liu B., Zhou H., Tan L., Siu K.T.H., Guan X.-Y. (2024). Exploring treatment options in cancer: Tumor treatment strategies. Signal Transduct. Target. Ther..

[7] Overcast W.B., Davis K.M., Ho C.Y., Hutchins G.D., Green M.A., Graner B.D., Veronesi M.C. (2021). Advanced imaging techniques for neuro-oncologic tumor diagnosis, with an emphasis on PET-MRI imaging of malignant brain tumors. Curr. Oncol. Rep..

[8] Jeljeli S., Cook G., Hammers A., Chiribiri A., Rogers H., Kinsella S., Krokos G., Neji R. (2025). Foundations of PET/MR. Nuclear Medicine Hybrid Imaging for Radiographers & Technologists.

[9] Vankdothu R., Hameed M.A. (2022). Brain tumor MRI images identification and classification based on the recurrent convolutional neural network. Meas. Sens..

[10] Saeedi S., Rezayi S., Keshavarz H., Niakan Kalhori S.R. (2023). MRI-based brain tumor detection using convolutional deep learning methods and chosen machine learning techniques. BMC Med. Inform. Decis. Mak..

[11] Kokila S., Praveena S. (2024). Combining A Hybrid Genetic Algorithm with A Fuzzy Logic Classifier Enhances Heart Disease Diagnosis. PatternIQ Min..

[12] Aamir M., Rahman Z., Dayo Z.A., Abro W.A., Uddin M.I., Khan I., Imran A.S., Ali Z., Ishfaq M., Guan Y. (2022). A Deep Learning Approach for Brain Tumor Classification Using MRI Images. Comput. Electr. Eng..

[13] Thenuwara G., Curtin J., Tian F. (2023). Advances in Diagnostic Tools and Therapeutic Approaches for Gliomas: A Comprehensive Review. Sensors.

[14] Ji D., Luo Z., Ovcjak A., Alanazi R., Bao M.-H., Feng Z.-P., Sun H.-S. (2021). Role of TRPM2 in Brain Tumours and Potential as a Drug Target. Acta Pharmacol. Sin..

[15] Zeng W.-J., Zhang L., Cao H., Li D., Zhang H., Xia Z., Peng R. (2022). A Novel Inflammation-Related LncRNAs Prognostic Signature Identifies LINC00346 in Promoting Proliferation, Migration, and Immune Infiltration of Glioma. Front. Immunol..

[16] Yang Y., Schubert M.C., Kuner T., Wick W., Winkler F., Venkataramani V. (2022). Brain Tumor Networks in Diffuse Glioma. Neurother. J. Am. Soc. Exp. Neurother..

[17] Qian K., Yao C., Wang Y., Yang Q., Xiang S., Pei Q., Zhu T., Liu H., Dong S. (2025). Potential of Ultrashort Pulsed Electric Fields to Disrupt Dense Structure in Glioma Tumors. IEEE Trans. Biomed. Eng..

[18] Lei Y., Dong S., Liang R., Xiang S., Huang Q., Ma J., Kou H., Yu L., Yao C. (2024). Parallel Resonant Magnetic Field Generator for Biomedical Applications. IEEE Trans. Biomed. Circuits Syst..

[19] Das S., Goswami R.S. (2024). Advancements in Brain Tumor Analysis: A Comprehensive Review of Machine Learning, Hybrid Deep Learning, and Transfer Learning Approaches for MRI-Based Classification and Segmentation. Multimed. Tools Appl..

[20] Shamshad N., Sarwr D., Almogren A., Saleem K., Munawar A., Rehman A.U., Bharany S. (2024). Enhancing Brain Tumor Classification by a Comprehensive Study on Transfer Learning Techniques and Model Efficiency Using MRI Datasets. IEEE Access.

[21] Yin L., Wang L., Lu S., Wang R., Ren H., AlSanad A., AlQahtani S.A., Yin Z., Li X., Zheng W. (2024). AFBNet: A Lightweight Adaptive Feature Fusion Module for Super-Resolution Algorithms. Comput. Model. Eng. Sci..

[22] Tabatabaei S., Rezaee K., Zhu M. (2023). Attention Transformer Mechanism and Fusion-Based Deep Learning Architecture for MRI Brain Tumor Classification System. Biomed. Signal Process. Control.

[23] Hekmat A., Zhang Z., Rehman Khan S.U., Bilal O. (2025). Brain Tumor Diagnosis Redefined: Leveraging Image Fusion for MRI Enhancement Classification. Biomed. Signal Process. Control.

[24] Jiang R., Yin X., Yang P., Cheng L., Hu J., Yang J., Wang Y., Fu X., Shang L., Li L. (2024). A Transformer-Based Weakly Supervised Computational Pathology Method for Clinical-Grade Diagnosis and Molecular Marker Discovery of Gliomas. Nat. Mach. Intell..

[25] Song Z., Yang B. (2022). Ant Colony Based Fish Crowding Degree Optimization Algorithm for Magnetic Resonance Imaging Segmentation in Sports Knee Joint Injury Assessment. Expert Syst..

[26] Zhu J., Gu C., Wei L., Li H., Jiang R., Sheykhahmad F.R. (2024). Brain Tumor Recognition by an Optimized Deep Network Utilizing Ammended Grasshopper Optimization. Heliyon.

[27] Daoud S., Nasayreh A., Nahar K.M.O., Abedalaziz W.K., Alayasreh S.M., Gharaibeh H., Bashkami A., Jaradat A., Jarrar S., Al-Hawamdeh H. (2025). A Novel Deep Learning-Based Spider Wasp Optimization Approach for Enhancing Brain Tumor Detection and Physical Therapy Prediction. Comput. Methods Programs Biomed. Updat..

[28] Meenal T., Asokan R. (2026). Quantum-Inspired Adaptive Feature Fusion for Highly Accurate Brain Tumor Classification in MRI Using Deep Learning. Biomed. Signal Process. Control.

[29] Yin L., Teng J. (2025). Rejection Recognition Deep Fusion Method of ResNet-Attention-EfficientNet-B0-Transformer for Brain Tumor Classification. Biomed. Signal Process. Control.

[30] Barati B., Erfaninejad M., Khanbabaei H. (2024). Evaluation of Effect of Optimizers and Loss Functions on Prediction Accuracy of Brain Tumor Type Using a Light Neural Network. Biomed. Signal Process. Control.

[31] Ullah M.S., Khan M.A., Albarakati H.M., Damaševičius R., Alsenan S. (2024). Multimodal Brain Tumor Segmentation and Classification from MRI Scans Based on Optimized DeepLabV3+ and Interpreted Networks Information Fusion Empowered with Explainable AI. Comput. Biol. Med..

[32] Saraswat M., Dubey A. (2025). kumar Early Stage Brain Tumor Prediction Using Dilated and Attention-Based Ensemble Learning with Enhanced Artificial Rabbit Optimization for Brain Data. Biomed. Signal Process. Control.

[33] Mallouk O., Joudar N.-E., Ettaouil M. (2025). ODTL: An Optimal Deep Transfer Learning Model for Brain Tumor Classification. Neurocomputing.

[34] Bernard D., Msigwa C., Yun J. (2025). Enhanced Magnetic Resonance Imaging Feature Extraction for Precise Brain Tumor Classification Using Dual Deep Convolutional Networks. Knowl.-Based Syst..

[35] Guo B., Huang W., Wang X. (2025). ABE-Mamba: Few-Shot Medical Image Segmentation via Adversarial Bidirectional Enhanced Mamba. Expert Syst. Appl..

[36] Wang W., Chen C., Ding M., Yu H., Zha S., Li J., de Bruijne M., Cattin P.C., Cotin S., Padoy N., Speidel S., Zheng Y., Essert C. (2021). TransBTS: Multimodal Brain Tumor Segmentation Using Transformer BT-Medical Image Computing and Computer Assisted Intervention—MICCAI 2021. Proceedings of the Lecture Notes in Computer Science.

[37] Chen Y., Jiang M., Xia C., Zhao H., Ke P., Chen S., Ge H., Li K., Wang X., Wang Y. (2025). A Novel Deep Learning System for STEMI Prognostic Prediction from Multi-Sequence Cardiac Magnetic Resonance. Sci. Bull..

[38] Hasan M.S., Rahman M., Fahim F., Islam J., Pervin T., Hasan M.M. (2025). DEEP Q-NAS: A New Algorithm Based on Neural Architecture Search and Reinforcement Learning for Brain Tumor Identification from MRI. Comput. Biol. Med..

[39] BRATS 2018. https://www.kaggle.com/datasets/sanglequang/brats2018.

[40] BRATS 2019. https://www.kaggle.com/datasets/aryashah2k/brain-tumor-segmentation-brats-2019.

[41] BRATS 2020. https://www.kaggle.com/datasets/awsaf49/brats20-dataset-training-validation.

[42] Figshare. https://www.kaggle.com/datasets/ashkhagan/figshare-brain-tumor-dataset.

